# *Gadd45a* knockout alleviates cisplatin-induced hearing loss by inhibiting CXCL family protein expression

**DOI:** 10.1016/j.mmr.2026.100063

**Published:** 2026-08-14

**Authors:** Wei-Long Wang, Sheng-Yu Zou, Dan-Qi Wang, Yue Liu, Jia-Wen Li, Fang-Zi Ke, Si-Hui Wen, Bo-Wen Xu, Kun Lin, Chun-Jiang Wei, Xiao-Long Fu, Qiao-Jun Fang, Xiao-Xiang Xu, Xiong Chen, Zu-Hong He

**Affiliations:** a Department of Otorhinolaryngology-Head and Neck Surgery, Zhongnan Hospital of Wuhan University, Wuhan 430071, China; b Medical Science and Technology Innovation Center, Institute of Brain Science and Brain-inspired Research, Shandong Provincial Hospital, Shandong First Medical University & Shandong Academy of Medical Sciences, Jinan 250117, China; c Department of Otolaryngology-Head and Neck Surgery, the Second Affiliated Hospital of Anhui Medical University, Hefei 230601, China; d Department of Otolaryngology Head and Neck Surgery, Zhongda Hospital, School of Medicine, Advanced Institute for Life and Health, Southeast University, Nanjing 210096, China; e Department of Otolaryngology, the First Affiliated Hospital of Wenzhou Medical University, Wenzhou, Zhejiang 325000, China

**Keywords:** Ototoxicity, Cisplatin, Growth arrest and DNA damage-inducible alpha (GADD45A), NF-κB1, CXCL family

## Abstract

**Background:**

Cisplatin is a widely used chemotherapeutic agent, but its clinical application is limited by serious ototoxic side effects, including hearing loss, tinnitus, and vertigo. Inflammation is now recognized as a significant contributor to cisplatin-induced hearing loss, but the upstream regulators remain largely undefined. This study aimed to identify novel regulators of cisplatin-induced ototoxicity through CRISPR-Cas9 screening and to determine the role of growth arrest and DNA damage-inducible alpha (GADD45A) in cochlear inflammation and hearing loss.

**Methods:**

To identify upstream regulators of cisplatin-induced ototoxicity, a CRISPR-Cas9-based loss-of-function screen was first performed in OC1 cells. Following the identification of *Gadd45a* as a candidate gene, its effects on cisplatin-induced cytotoxicity were evaluated using cell viability assays, flow cytometry, and TUNEL staining. Integrated transcriptomic and proteomic analyses were subsequently conducted to elucidate the downstream molecular mechanisms. Finally, the protective role of *Gadd45a* inhibition was validated *in vivo* through siRNA delivery into the posterior semicircular canal and in *Gadd45a* conditional knockout (cKO) mice.

**Results:**

*Gadd45a* knockout significantly inhibited cisplatin-induced cell death in the OC1 cell line while simultaneously enhancing autophagy signaling. Transcriptomic profiling revealed a marked downregulation of the expression of C-X-C motif chemokine ligand (CXCL) family cytokines following *Gadd45a* loss, which was further supported by proteomic analysis of culture supernatants. Mechanistically, *Gadd45a* knockout specifically inhibited nuclear factor κB subunit 1 (NF-κB1) but not RELA proto-oncogene, NF-κB subunit (RELA). Immunofluorescence staining demonstrated that *Gadd45a* knockout inhibited the nuclear translocation of the NF-κB1 protein, and subsequent studies revealed that the NF-κB1 protein was degraded through the lysosomal pathway. *In vivo*, both si-*Gadd45a* injection into the semicircular canal and the knockout of *Gadd45a* effectively attenuated cisplatin-induced ototoxicity.

**Conclusions:**

This study identifies GADD45A as a key regulator of cisplatin ototoxicity and links the balance between apoptosis and autophagy with NF-κB1-CXCL-mediated inflammation. Targeted inhibition of GADD45A signaling may represent a promising therapeutic strategy to prevent hearing loss in patients receiving cisplatin chemotherapy.

## 1 Background

Cisplatin is a highly effective, broad-spectrum antitumor drug that is commonly used in clinical settings [1] and is effective against a variety of solid tumors [2]. The anticancer mechanism of cisplatin relies on DNA cross-linking, which disrupts DNA replication and transcription factor binding, leading to dysregulated gene expression and cell death [3]. However, clinical application has revealed that cisplatin has serious ototoxic side effects, which typically manifest as bilateral, irreversible sensorineural hearing loss [4] and tinnitus [5]. As the outer hair cells (OHCs) of the cochlear base are vulnerable to these effects, hearing loss first occurs in the high-frequency range [6]. With the accumulation of cisplatin in the cochlear tissue, hearing loss occurs in the middle- and low-frequency ranges and may eventually lead to full-frequency hearing loss [7]. Cisplatin can also damage various structures in the inner ear, including hair cells, the stria vascularis, the basilar membrane, the osseous spiral lamina, spiral ligaments, and spiral ganglion cells [8]. As a consequence, cisplatin-induced ototoxicity causes permanent hearing loss in approximately 60% of adult patients and at least half of pediatric patients [9], [10], [11]. Sodium thiosulfate has recently been approved by the US Food and Drug Administration (FDA) for otoprotection in pediatric patients with local nonmetastatic solid tumors [12]. It only partially reduces high-frequency hearing loss and has no significant protective effect on low-frequency hearing loss. Currently, there are no drugs that inhibit the ototoxicity of cisplatin in adult patients or children with metastatic cancer [13].

Growth arrest and DNA damage-inducible alpha (GADD45A) is involved in stress signaling pathways triggered by physiological and environmental stressors, and participates in multiple cellular processes, including DNA repair, cell-cycle regulation, and stress kinase signaling [14], [15]. During acute stress and inflammation, GADD45A contributes to the chemotactic response of immune cells to lipopolysaccharide (LPS) and other inflammatory stimuli and modulates myeloid-cell responses [16], [17], [18]. *Gadd45a* deficiency has also been associated with enhanced autophagic activity, characterized by increased microtubule-associated protein 1 light chain 3-II (LC3-II) expression and reduced sequestosome 1 (SQSTM1) levels through modulation of the beclin 1-phosphatidylinositol 3-kinase catalytic subunit type 3 (BECN1-PIK3C3) complex [19], suggesting that the biological functions of GADD45A are context-dependent and may influence both cell survival and cell death. Previous studies have reported that both intense noise [20] and high doses of aspirin [21] significantly upregulate GADD45A expression in the inner ear, suggesting a potential role in hearing loss. Given the protective role of autophagy against cisplatin-induced apoptosis [22], these findings suggest that GADD45A may influence cellular susceptibility to stress-induced injury.

Although the molecular mechanisms underlying cisplatin-induced ototoxicity remain incompletely understood, accumulating evidence suggests that inflammation plays a central role in both processes. Cytokines and chemokines are now recognized as key mediators of cisplatin-induced hearing loss [23], [24]. For instance, after cisplatin treatment, the migration of immune cells expressing CD45, CD68, and ionized calcium-binding adaptor molecule 1 (IBA1) into the cochlea results in the accumulation of the cytokine C-X-C motif chemokine ligand 1 (CXCL1) in spiral ganglion neurons (SGNs) and the organ of Corti in a time-dependent manner. Intratympanic injection of SB225002, a chemical inhibitor targeting the CXCL1 receptor (CXCR2), can protect against cisplatin-induced hair cell loss and hearing impairment [25], [26], [27]. Nuclear factor κB (NF-κB) is a key transcription factor regulating the expression of multiple inflammatory chemokines, including members of the CXCL family. GADD45A mediates p38 phosphorylation and the degradation of inhibitor of nuclear factor κB (IκB), an inhibitor of the NF-κB signaling pathway, leading to NF-κB translocation to the nucleus for the transcription of downstream genes [28]. Moreover, NF-κB1 can stabilize GADD45A through ubiquitination and proteasome-dependent degradation and subsequently activate the mitogen-activated protein kinase kinase 4-c-Jun N-terminal kinase (MKK4-JNK) signaling pathway and induce apoptosis [29]. However, whether GADD45A contributes to cisplatin-induced cochlear inflammation through NF-κB1-dependent inflammatory signaling remains unclear.

Although siGadd45a has shown neuroprotective potential in hippocampal neurons by attenuating neuronal injury and improving functional outcomes [30], the specific regulatory mechanisms of GADD45A in cisplatin-induced ototoxicity and its link to the inflammatory response require further elucidation. The primary objective of this study was to investigate the role of GADD45A in cisplatin-induced ototoxicity and elucidate the underlying molecular mechanisms. Specifically, we sought to elucidate the molecular mechanisms underlying GADD45A-mediated cochlear inflammation and to evaluate its therapeutic potential as a target for preventing cisplatin-induced hearing loss.

## 2 Methods

### 2.1 Animals

C57BL/6 J mice (male, 7 weeks old) were purchased from SPF Biotech (Beijing, China). *Gadd45a* conditional knockout (cKO) mice were obtained from Cyagen Biosciences, Inc. (Suzhou, China). GFI^Cre^ mice were obtained from The Jackson Laboratory (Bar Harbor, ME, USA). All the mice were housed in a standard environment with a temperature of 22 °C under a 12/12 h light-dark cycle and a constant humidity of 50%–60%. The mice had free access to food and water. The cisplatin used for animal treatment was pharmaceutical-grade cisplatin for injection, obtained from Qilu Pharmaceutical (Hainan, China) Co., Ltd. (specification: 20 mg). The methods and primers used for mouse genotyping used in this experiment are shown in Additional file 1: Table S1. All animal procedures were approved by the Laboratory Animal Welfare & Ethics Committee of Zhongnan Hospital of Wuhan University (WP20240254) and were performed in accordance with the relevant institutional guidelines.

### 2.2 Gene knockout cell line construction and cell culture

The construction of gene knockout cell lines was performed as previously described in our earlier studies [31], [32], [33]. The immortalized OC1 cell line, which expresses multiple hair cell markers originating from the cochlear sensory epithelium, was utilized in our experiments. This cell line has been used in several previous studies [34], [35], [36], [37], [38], [39]. The OC1 cell line was cultured in high-glucose DMEM (Gibco, Life Technologies, Australia Pty Ltd., Australia) supplemented with 10% fetal bovine serum (F8318, Sigma, Darmstadt, Germany) in a 5% CO₂ incubator at 37 °C. The sequences of the siRNAs and primers used in this experiment are shown in Additional file 1: Tables S2 and S3.

### 2.3 Auditory brainstem response test

The auditory brainstem response (ABR) test serves as an objective method for measuring hearing thresholds. Specifically, normal hearing ability was ensured before each treatment, as previously described [40], [41]. We first measured the body weights of the mice, after which ketamine (100 mg/kg) and xylazine (25 mg/kg) were injected intraperitoneally to anesthetize the mice. Following satisfactory anesthesia induction (as indicated by hand-pinching mouse toes without painful reflex movements), recording electrodes were inserted subcutaneously. The hearing thresholds of the mice were then tested at 8, 16, and 32 kHz frequencies and clicks using a Tucker-Davis Technologies (TDT) RZ6 system (Alachua, FL, USA).

### 2.4 RNA-sequencing, cell culture supernatant proteome-sequencing, and bioinformatic analyses

OC1 and *Gadd45a*^-/-^ cells were seeded separately in 10 cm plates and harvested upon reaching 90%–95% confluence. The cells were subjected to transcriptome sequencing, and the cell culture supernatant was used for proteome sequencing at Novogene Biotechnology Co. Ltd. (Beijing, China). Differentially expressed genes were identified based on the sequencing results. Gene Ontology (GO) enrichment analysis and Kyoto Encyclopedia of Genes and Genomes (KEGG) pathway analysis were subsequently performed for the differentially expressed genes to identify significantly enriched biological processes and signaling pathways. Terms and pathways with an adjusted P-value<0.05 were considered statistically significant. The JASPAR database (https://jaspar.elixir.no) was searched to assess whether GADD45A has a reported transcription factor-binding profile and to predict potential shared transcription factors regulating CXCL1, CXCL3, and CXCL10.

### 2.5 Immunofluorescence staining

The mice were sacrificed following ABR testing. The cochlea was then removed, and a hole was made in the apex of the cochlea. Paraformaldehyde (PFA) (4%) was slowly injected into the cochlea through the oval window using a 1 ml syringe. The outflow of PFA from the apex indicated that the entire cochlea was filled. After 1.5 h of fixation in 4% PFA on a shaker at room temperature, the cochleae were decalcified in 10% EDTA solution at 4 °C on a shaker. The EDTA solution was refreshed daily for 4 consecutive d. Following decalcification, the auditory epithelium was divided into two sections (apex-middle and base-hook) using ophthalmic scissors (Jinzhong Surgical Instrument, China). The cochlear basilar membranes were carefully isolated with dissecting forceps (Dumont, Switzerland) and pathology scalpels (Feather, Japan). The isolated cochlear basilar membranes were then allowed to adhere to 9-mm diameter coverslips with Cell-Tak (354240; Corning, USA), perforated with 2% Triton X-100 for 15 min at room temperature, and then blocked with 10% donkey serum at room temperature for 1 h. The membranes were subsequently washed three times with phosphate-buffered saline (PBS) for 5 min each and then incubated overnight with primary antibodies. The information on the antibodies used in this study is provided in Additional file 1**:** Table S4.

To calculate the number of cochlear hair cells lost, the samples were incubated with an anti-myosin VIIa antibody (#25-6790; 1:1000, Proteus Biosciences, USA) overnight at 4 °C. After three washes with PBS, the samples were stained with goat anti-rabbit IgG conjugated to Alexa Fluor 488 (A-11008; 1:500, Invitrogen, Thermo Fisher Scientific, USA) and phalloidin conjugated to Alexa Fluor 594 (A-12381; 1:500, Invitrogen, Thermo Fisher Scientific, USA) overnight at 4 °C. Following three additional washes with PBS, the nuclei were stained with DAPI (10236276001; Roche, Switzerland) diluted 1:1000 for 30 min at room temperature. Phalloidin-labeled hair cell stereocilia and myosin VIIa-labeled cell cytoplasm were observed under a fluorescence confocal microscope using appropriate filters.

To examine the synapses of inner hair cells (IHCs), the cochlear basilar membranes were first isolated and then treated with 3% Triton X-100 for 30 min at room temperature to permeabilize the tissues. The membranes were subsequently blocked with 10% donkey serum at room temperature for 1 h. After blocking, the samples were washed three times with PBS for 5 min each. The samples were then incubated overnight at 37 °C with primary antibodies, including an anti-C-terminal binding protein 2 (CtBP2) IgG1 antibody (#612044; 1:200, BD Biosciences, USA) and an anti-AMPA-type glutamate receptor subunit 2 (GluR2) IgG2a antibody (#MAB397; 1:1000, Millipore, USA). The samples were subsequently washed three times with PBS for 5 min each. Next, the samples were incubated at 37 °C for 1 h with the following secondary antibodies: Alexa Fluor 594-conjugated goat anti-mouse IgG1 (A-21125; 1:1000, Invitrogen, USA) and Alexa Fluor 488-conjugated goat anti-mouse IgG2a (A-21131; 1:1000, Invitrogen, USA). This secondary antibody incubation was repeated for 1 h at 37 °C. After synaptic staining, the hair cells were stained with an anti-myosin VIIa antibody (#25-6790; 1:1000, Proteus Biosciences, USA) overnight at 4 °C. Finally, the cochlear basilar membranes were washed three times with PBS for 5 min each and incubated overnight at 4 °C with a secondary antibody: Alexa Fluor 647-conjugated goat anti-rabbit IgG (A-21235; 1:1000, Invitrogen, USA). Finally, all cochlear basilar membranes were washed three times with PBS for 5 min each, and 15 μl Dako mounting medium (Dako, Denmark) was added to each coverslip, which was then covered with another coverslip. The edges were sealed with nail polish. The cochlear basilar membrane was photographed using a confocal microscope (Leica TCS SP8, Leica Microsystems GmbH, Wetzlar, Germany).

### 2.6 Neonatal cochlear membrane culture

Cochlear organotypic cultures were prepared from postnatal day 3 (P3) SD rat pups as described in our previous publications [42], [43], [44]. Following incubation of the cochlear membranes with cisplatin for 24 and 48 h, three cochlear membranes were harvested using dissecting forceps and placed into a 1.5 ml Eppendorf tube as one sample. Subsequently, 200 μl of RIPA lysis buffer (P0013B; Beyotime Biotech Inc., China) supplemented with protease inhibitor cocktail tablets (04693159001; Roche, Switzerland) was added to the tube. The samples were then homogenized using a freezing-type grinding instrument and subjected to Western blotting analysis.

### 2.7 Injection of siRNA into the inner ear through posterior semicircular canal

To evaluate the stability and retention time of siRNA in the inner ear, 5’-fluorescein amidite-labeled siRNA (5’-FAM-siRNA), with an excitation wavelength of 494 nm and an emission wavelength of 520 nm, was mixed with Invivofectamine 3.0 Reagent (Invitrogen, IVF3001, Thermo Fisher Scientific, USA) according to the complexation protocol. The siRNA was then injected into the inner ear via the posterior semicircular canal (PSC). An *in vivo* imaging system (IVIS SPECTRUM, PerkinElmer, USA) was used to evaluate the retention effect. Similarly, *Gadd45a* siRNA (catalog No. s64885; Thermo Fisher Scientific, USA) was injected into the left ear through the PSC for subsequent experiments.

Other experimental details are provided in the Additional file 1**:** Methods.

### 2.8 Statistical analysis

Statistical analyses were performed using GraphPad Prism software (version 9.0; La Jolla, CA, USA). All the data are presented as the mean±standard deviation (SD). A paired *t*-test was used to compare the data between two groups. One-way analysis of variance (ANOVA) was used to assess differences among multiple groups. Tukey’s multiple comparisons test was used to assess differences between groups. *P*-values of <0.05 were considered statistically significant.

## 3 Results

### 3.1 *Gadd45a* knockout inhibits cisplatin-induced cell death

**Fig. 1:**
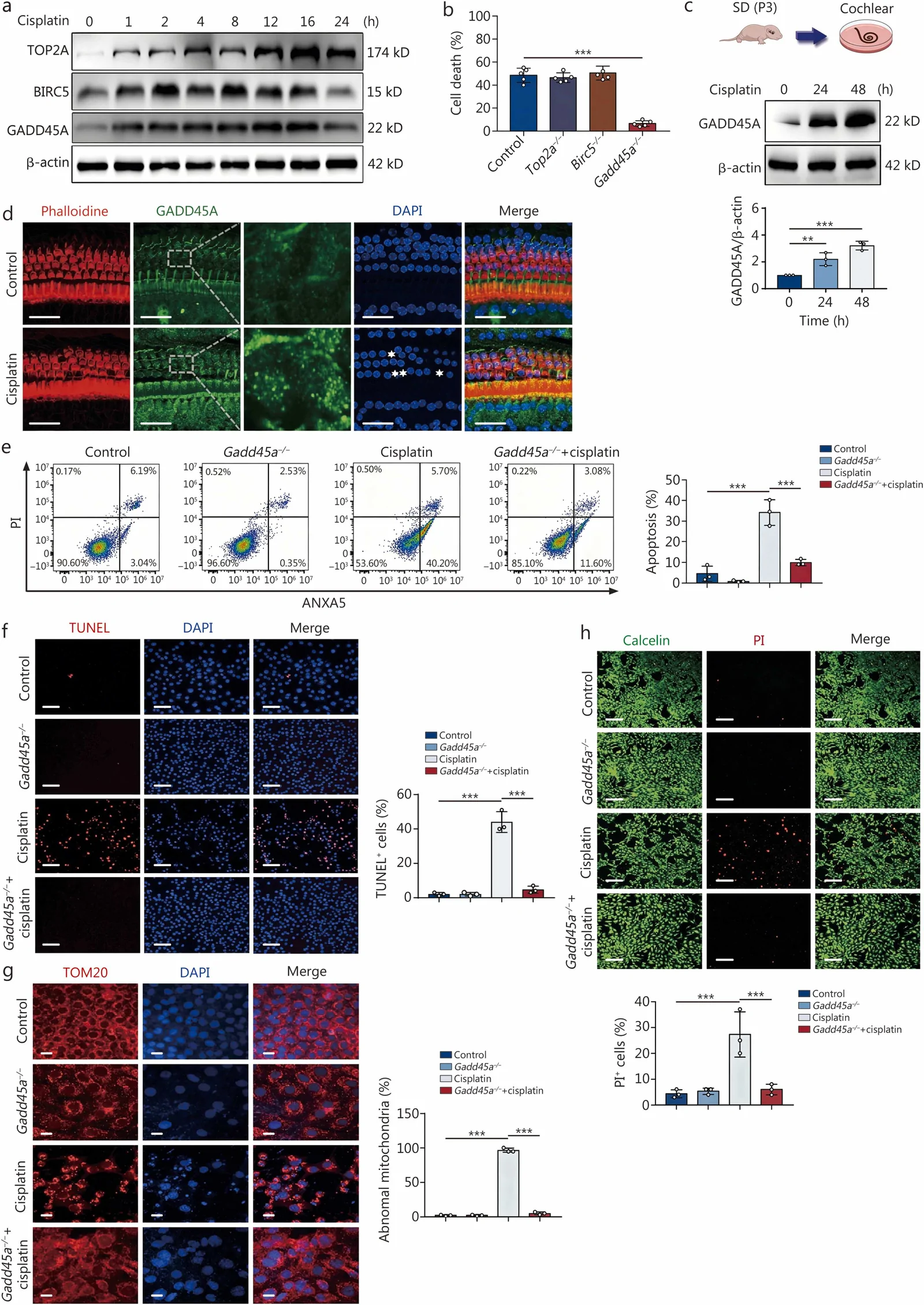
*Gadd45a* knockout inhibits cisplatin-induced apoptosis. a OC1 cells were treated with 25 μmol/L cisplatin for different durations, and TOP2A, BIRC5, and GADD45A protein expression levels were visualized by Western blotting analysis. b Cisplatin-induced cell death rates of the *Top2a*, *Birc5,* and *Gadd45a* knockout cell lines. c Schematic illustration of the SD (P3) neonatal rat cochlear explant culture model and representative Western blotting analysis of GADD45A expression following treatment with 25 μmol/L cisplatin for 24 and 48 h. d Immunofluorescence staining of GADD45A protein expression in hair cells after intraperitoneal injection of cisplatin in adult C57BL/6 J mice. Asterisks indicate missing hair cells. Scale bar=20 μm. e Representative flow cytometry plots and statistical analysis of early apoptotic cells in control and *Gadd45a* knockout cells with or without 25 μmol/L cisplatin treatment for 24 h. f Representative images of TUNEL staining and statistical analysis of TUNEL^+^ cells in control and *Gadd45a* knockout cells following treatment with 25 μmol/L cisplatin for 24 h. Scale bar=100 μm. g Representative images of TOM20 staining and quantification of cells with abnormal mitochondrial morphology in control and *Gadd45a* knockout cells following treatment with 25 μmol/L cisplatin for 24 h. Scale bar=20 μm. h Representative images of Live/Dead staining and quantification of PI^+^ cells in control and *Gadd45a* knockout cells following treatment with 25 μmol/L cisplatin for 24 h. Scale bar=100 μm. ^∗∗^*P*<0.01, ^∗∗∗^*P*<0.001; horizontal bars indicate the groups compared. TOP2A. DNA topoisomerase II alpha; BIRC5. Baculoviral inhibitor of apoptosis repeat-containing 5; GADD45A. Growth arrest and DNA damage-inducible alpha; SD. Sprague–Dawley; P3. Postnatal day 3; PI. Propidium iodide; TUNEL. Terminal deoxynucleotidyl transferase-mediated dUTP nick end labeling; TOM20. Translocase of outer mitochondrial membrane 20; DAPI. 4’,6-diamidino-2-phenylindole.

Based on DNA topoisomerase II alpha (TOP2A), baculoviral IAP repeat-containing 5 (BIRC5), and GADD45A in platinum resistance and cell survival pathways [45], [46], [47], [48], we measured TOP2Α, BIRC5, and GADD45A protein expression levels after treatment with 25 μmol/L cisplatin at various time points in OC1 cells. All this showed a similar pattern, TOP2Α and GADD45A by an initial increase followed by a decrease, and an increasing trend in BIRC5 (Fig. 1**a,** Additional file 1**:** Fig. S1a). To determine whether *Top2a*, *Birc5*, and *Gadd45a* genes contribute to cisplatin-induced cell death, we knocked out the expression of these genes using CRISPR-Cas9 and verified the knockouts by Western blotting, confirming the knockout of expression of each gene (Additional file 1**:** Fig. S1b). We then assessed cell viability using a CCK-8 assay in *Top2a*^*-/-*^, *Birc5*^*-/-*^, and *Gadd45a*^*-/-*^ cells following cisplatin treatment. *Top2a* or *Birc5* knockout did not significantly affect cell viability after cisplatin treatment, whereas *Gadd45a* knockout significantly increased cell viability (Fig. 1**b**).

Based on this protective phenotype, we selected GADD45A as a molecular target for further investigation. Additionally, cochlear organotypic cultures derived from SD rat pups were treated with cisplatin for 24 and 48 h. Cisplatin treatment increased GADD45A protein expression (Fig. 1**c**). To further explore the role of GADD45A expression in cisplatin-induced cochlear hair cell death *in vivo*, adult C57BL/6 J mice were intraperitoneally injected with cisplatin at a dose of 4 mg/kg, as described in previous studies [5]. After cisplatin treatment, GADD45A protein expression was found to be elevated by immunofluorescence analysis (Fig. 1**d**). Consistent with the initial screening results, *Gadd45a*^−/−^ cells exhibited greater resistance to cisplatin across a range of concentrations, maintaining significantly higher viability than control cells (Additional file 1**:** Fig. S2a).

Flow cytometry revealed that the proportion of early apoptotic cells decreased from 34.13% in the cisplatin group to 9.87% in the *Gadd45a*^−/−^ group following cisplatin treatment (Fig. 1**e**). TUNEL staining further confirmed these findings, with significantly fewer TUNEL^+^ cells observed in the *Gadd45a*^−/−^ group following cisplatin treatment than in the cisplatin group (Fig. 1**f**). Furthermore, translocase of outer mitochondrial membrane 20 (TOM20), a receptor subunit of the TOM complex involved in mitochondrial protein transport, exhibited marked aggregation and abnormal distribution following cisplatin treatment, whereas these alterations were substantially attenuated in *Gadd45a*^−/−^ cells exposed to cisplatin (Fig. 1**g**). Live/dead staining further confirmed these findings, with numerous propidium iodide-positive (PI^+^) dead cells observed in the cisplatin-treated control group, whereas markedly fewer dead cells were detected in the *Gadd45a*^−/−^ group following cisplatin treatment (Fig. 1**h**). Statistical analysis revealed that the percentage of PI^+^ cells decreased from approximately 27.33% in the cisplatin group to about 5.66% in the *Gadd45a*^−/−^ group following cisplatin treatment (Fig. 1**h**). Finally, mitochondrial reactive oxygen species (ROS) levels were assessed using MitoSOX red and compared with the cisplatin group, the *Gadd45a*^−/−^ group following cisplatin treatment exhibited markedly reduced ROS accumulation (Additional file 1: Fig. S2b).

### 3.2 *Gadd45a* knockout activates autophagy signaling

**Fig. 2:**
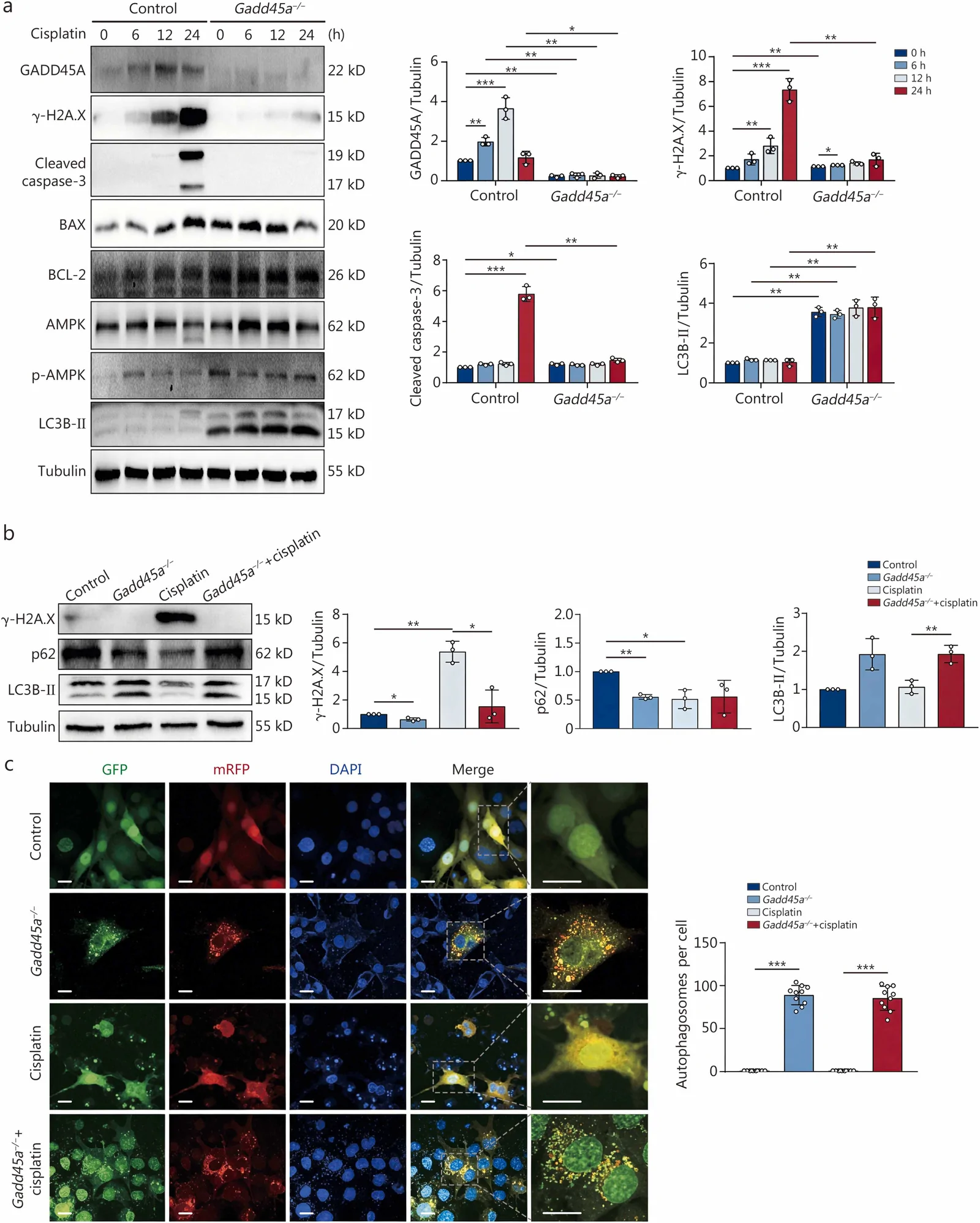
*Gadd45a* knockout activates autophagy signaling. Representative Western blotting images and quantification of GADD45A, γ-H2A.X, cleaved caspase-3, and LC3B-II expression in control and *Gadd45a* knockout cells following treatment with 25 μmol/L cisplatin for 0, 6, 12, and 24 h. **b** Representative Western blotting images and quantification of γ-H2A.X, p62, and LC3B-II expression in control and *Gadd45a* knockout cells following treatment with 25 μmol/L cisplatin for 24 h. **c** Representative images of tandem fluorescent mRFP-GFP-LC3 staining and quantification of autophagosomes (yellow puncta) in control and *Gadd45a* knockout cells following treatment with or without 25 μmol/L cisplatin for 24 h. Scale bar=20 μm.^∗^*P*<0.05, ^∗∗^*P*<0.01, ^∗∗∗^*P*<0.001; horizontal bars indicate the groups compared. GADD45A. Growth arrest and DNA damage-inducible alpha; γ-H2A.X. Phosphorylated H2A histone family member X; BAX. BCL-2-associated X protein; BCL-2. B-cell lymphoma-2; AMPK. AMP-activated protein kinase; p-AMPK. Phosphorylated AMP-activated protein kinase; LC3B-II. Microtubule-associated protein 1 light chain 3 beta-II; p62. Sequestosome 1; mRFP-GFP. Monomeric red fluorescent protein-green fluorescent protein; DAPI. 4’,6-diamidino-2-phenylindole.

To elucidate the mechanism by which *Gadd45a* knockout protects cells from cisplatin-induced toxicity, we collected protein samples from both the control and *Gadd45a*^*-/-*^ groups for Western blotting analysis at various time points after cisplatin treatment (Fig. 2**a**). The results demonstrated that the expression of phosphorylated H2A histone family member X (γ-H2A.X), a sensitive marker of DNA damage, progressively increased in the control group as the duration of cisplatin exposure increased, indicating increased DNA damage. In contrast, γ-H2A.X induction was markedly attenuated in the *Gadd45a*^*-/-*^ group. Additionally, the apoptosis-related protein, cleaved caspase-3, was significantly activated in the control group after 24 h of cisplatin treatment but remained inactive in the *Gadd45a*^*-/-*^ group. Consistent with reduced apoptosis, cleaved caspase-3 and BCL2-associated X protein (BAX) expression increased following cisplatin treatment in control cells but showed minimal changes in *Gadd45a*^−/−^ cells. In contrast, B-cell lymphoma 2 (BCL-2) expression was significantly higher in *Gadd45a*^−/−^ cells than in control cells (Additional file 1: Fig. S2c). Notably, the expression of AMP-activated protein kinase (AMPK) and phosphorylated AMPK (p-AMPK) was significantly elevated in the *Gadd45a*^*-/-*^ group. To further assess the autophagic activity in *Gadd45a*^−/−^ cells, we examined the expression of LC3B-II and SQSTM1 (p62). Compared with control cells, *Gadd45a*^−/−^ cells exhibited increased LC3B-II expression and reduced SQSTM1 (p62) levels, supporting enhanced autophagic activity (Fig. 2**b**). Moreover, immunofluorescence staining revealed a marked increase in the number of autophagosomes in *Gadd45a*^−/−^ cells, particularly following cisplatin treatment, supporting enhanced autophagic activity (Fig. 2**c**).

### 3.3 *Gadd45a* knockout downregulates the expression of CXCL family genes via NF-κB1 suppression

**Fig. 3:**
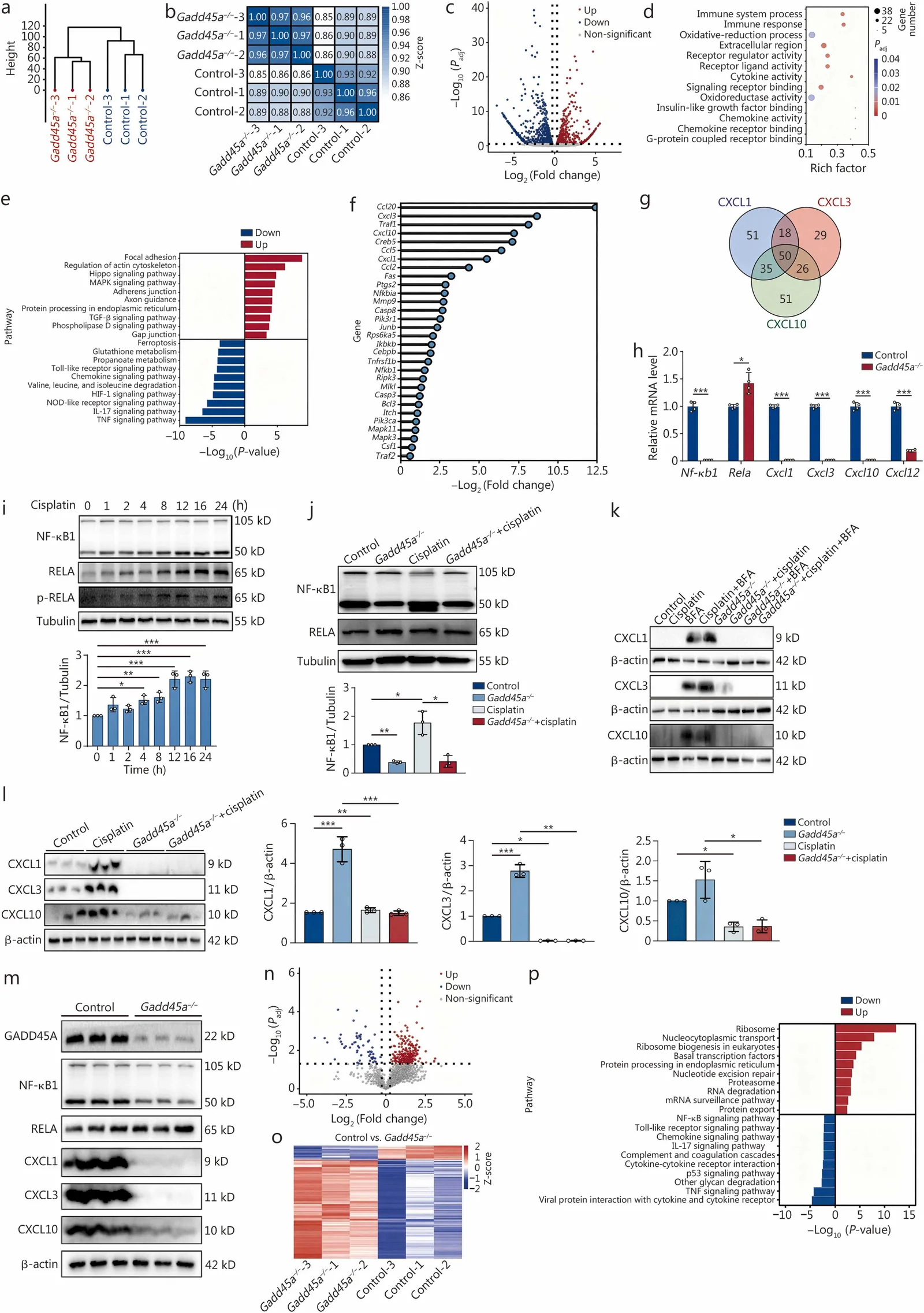
*Gadd45a* knockout downregulates CXCL family gene expression by decreasing expression of transcription factor NF-κB1. a Hierarchical clustering analysis of gene expression data. b Pearson correlation of transcriptome sequencing in control and *Gadd45a* knockout groups. c Volcano map of transcriptome sequencing in control and *Gadd45a* knockout groups. d Gene function analysis based on GO revealed the most altered pathways based on transcriptome sequencing in control and *Gadd45a* knockout groups. e Upregulation and downregulation of pathway activation are exhibited based on KEGG pathway analysis. f Ranking of genes related to the TNF signaling pathway. g Venn diagram of CXCL1/3/10 transcription factors. h Relative mRNA expression of *Nf-κb1*, *Rlea,* and *Cxcl1/3/10/12* to that of β-actin in control cells compared with *Gadd45a* knockout cells as determined by qRT-PCR. i Representative Western blotting images and quantification of NF-κB1, RELA, and p-RELA expression in OC1 cells following treatment with 25 μmol/L cisplatin for the indicated times. j Representative Western blotting images and quantification of NF-κB1 expression in control and *Gadd45a* knockout cells following treatment with 25 μmol/L cisplatin for 24 h. k The expression of CXCL1/3/10 proteins in the control group and *Gadd45a* knockout group after BFA or cisplatin alone or cotreatment was determined by Western blotting. l Representative Western blotting images and quantification of CXCL1, CXCL3, and CXCL10 expression in concentrated cell culture supernatants. m The expression levels of GADD45A, NF-κB1, RELA, CXCL1, CXCL3, and CXCL10 in control and *Gadd45a* knockout groups were detected by Western blotting. n Volcano map of protein sequencing in the control and *Gadd45a* knockout groups. o Heatmap of protein sequencing in the control and *Gadd45a* knockout groups. p Up and downregulation of pathway activation were exhibited between the control and *Gadd45a* knockout group. ^∗^*P*<0.05, ^∗∗^*P*<0.01, ^∗∗∗^*P*<0.001; horizontal bars indicate the groups compared. GADD45A. Growth arrest and DNA damage-inducible alpha; Gadd45a−/−. Gadd45a knockout; NF-κB1. Nuclear factor kappa B subunit 1; RELA. RELA proto-oncogene, NF-κB subunit; p-RELA. Phosphorylated RELA; CXCL. C-X-C motif chemokine ligand; KEGG. Kyoto Encyclopedia of Genes and Genomes; GO. Gene Ontology; BFA. Brefeldin A.

To investigate transcriptomic alterations associated with *Gadd45a* depletion, control and *Gadd45a*^*-/-*^ OC1 cells were subjected to transcriptome sequencing. Hierarchical clustering analysis demonstrated a clear separation between the two groups, indicating distinct transcriptional profiles (Fig. 3**a**). Correlation analysis showed higher intra-group correlation within each group and reduced similarity between *Gadd45a*^*-/-*^and control (Fig. 3**b**). Differential expression analysis was visualized using a volcano plot, which revealed a set of upregulated and downregulated genes in *Gadd45a*^*-/-*^ cells compared with control cells (Fig. 3**c**). GO enrichment analysis showed that differentially expressed genes were predominantly associated with immune-related biological processes (Fig. 3**d**). KEGG pathway analysis revealed that the downregulated genes were enriched in chemokine-related pathways, including tumor necrosis factor (TNF) and interleukin-17 (IL-17) signaling pathways (Fig. 3**e**). To further explore the specific genes contributing to this enrichment, we examined the differentially expressed gene set and identified multiple chemokine-related genes with marked changes, including members of the CXCL family (*Cxcl1, Cxcl3, and Cxcl10*) (Fig. 3**f**). Based on these gene-level alterations, we focused on the CXCL family as potential downstream targets of *Gadd45a* depletion.

To determine whether *Gadd45a* functions as a transcription factor, we searched for its binding motifs and structural domains in the JASPAR database. No DNA-binding motif or transcription factor-binding profile for GADD45A was identified in the JASPAR database. However, the coordinated downregulation of *Cxcl1/3/10* following *Gadd45a* knockout suggests the existence of a shared upstream transcriptional regulator. Analysis of predicted transcriptional regulators for CXCL1, CXCL3, and CXCL10 identified 50 shared transcription factors (Fig. 3**g**). Among the top 3 transcription factors ranked by their scores, all were related to the NF-κB signaling pathway, prompting us to focus on whether NF-κB mediates the *Gadd45a* knockout-induced reduction in CXCL family gene expression (Additional file 1**:** Fig. S3a). Correspondingly, we validated decreased mRNA expression of *Nf-κb1* and CXCL family genes after *Gadd45a* knockout, whereas *Rela* expression was increased (Fig. 3**h**). Similar trends were observed in cells treated with cisplatin for 24 h (Additional file 1**:** Fig. S3b). The protein expression of NF-κB1, RELA, and p-RELA also gradually increased after cisplatin treatment (Fig. 3**i;** Additional file 1**:** Fig. S3c). Notably, after 24 h of cisplatin treatment, *Gadd45a* knockout reduced NF-κB1 protein expression, whereas RELA expression remained unchanged (Fig. 3**j;** Additional file 1**:** Fig. S3d).

Considering that CXCL1/3/10 are secreted proteins and cannot be directly measured through cell lysis, cells were treated with brefeldin A (BFA) (Additional file 1**:** Fig. S3e) to block protein secretion and facilitate intracellular detection of CXCL proteins. Following BFA treatment, intracellular CXCL1, CXCL3, and CXCL10 proteins accumulated in control cells but remained barely detectable (Fig. 3**k;** Additional file 1**:** Fig. S3f), indicating that *Gadd45a* knockout inhibits CXCL protein expression. ELISA analysis revealed that CXCL1 secretion was markedly reduced in *Gadd45a*^−/−^ cells under both basal and cisplatin-treated conditions (Additional file 1**:** Fig. S3g).

To further evaluate secreted CXCL family proteins, concentrated culture supernatants and cell lysates were analyzed separately. CXCL1, CXCL3, and CXCL10 protein levels were increased in the supernatant of cisplatin-treated control cells. Still, they remained barely detectable in *Gadd45a*^−/−^ cells regardless of cisplatin treatment (Fig. 3**l**). Consistent with the preceding findings, *Gadd45a* knockout was associated with reduced NF-κB1 expression and concomitant decreases in CXCL1, CXCL3, and CXCL10 protein levels, whereas RELA expression remained largely unchanged (Fig. 3**m;** Additional file 1**:** Fig. S3h). To further characterize changes in the secreted proteome, culture supernatants were subjected to proteomic analysis. Volcano plot analysis identified CXCL1 as one of the most prominently altered proteins in the secretome (Fig. 3**n**). Heatmap analysis revealed distinct protein expression patterns between control and *Gadd45a*^−/−^ samples (Fig. 3**o**). A Venn diagram showed the distribution of functional annotation results across different databases. Functional annotation analysis revealed overlapping and database-specific annotations among GO, KEGG, eukaryotic orthologous groups (KOGs), and interPro (IPR) datasets (Additional file 1**:** Fig. S3i). KEGG pathway analysis revealed that the downregulated pathways were mainly related to viral protein interactions with cytokines and cytokine receptors, as well as the TNF signaling pathway (Fig. 3**p**). Gene set enrichment analysis (GSEA) revealed that extracellular protein enrichment decreased when *Gadd45a* was knocked out (Additional file 1**:** Fig. S3j).

**Fig. 4:**
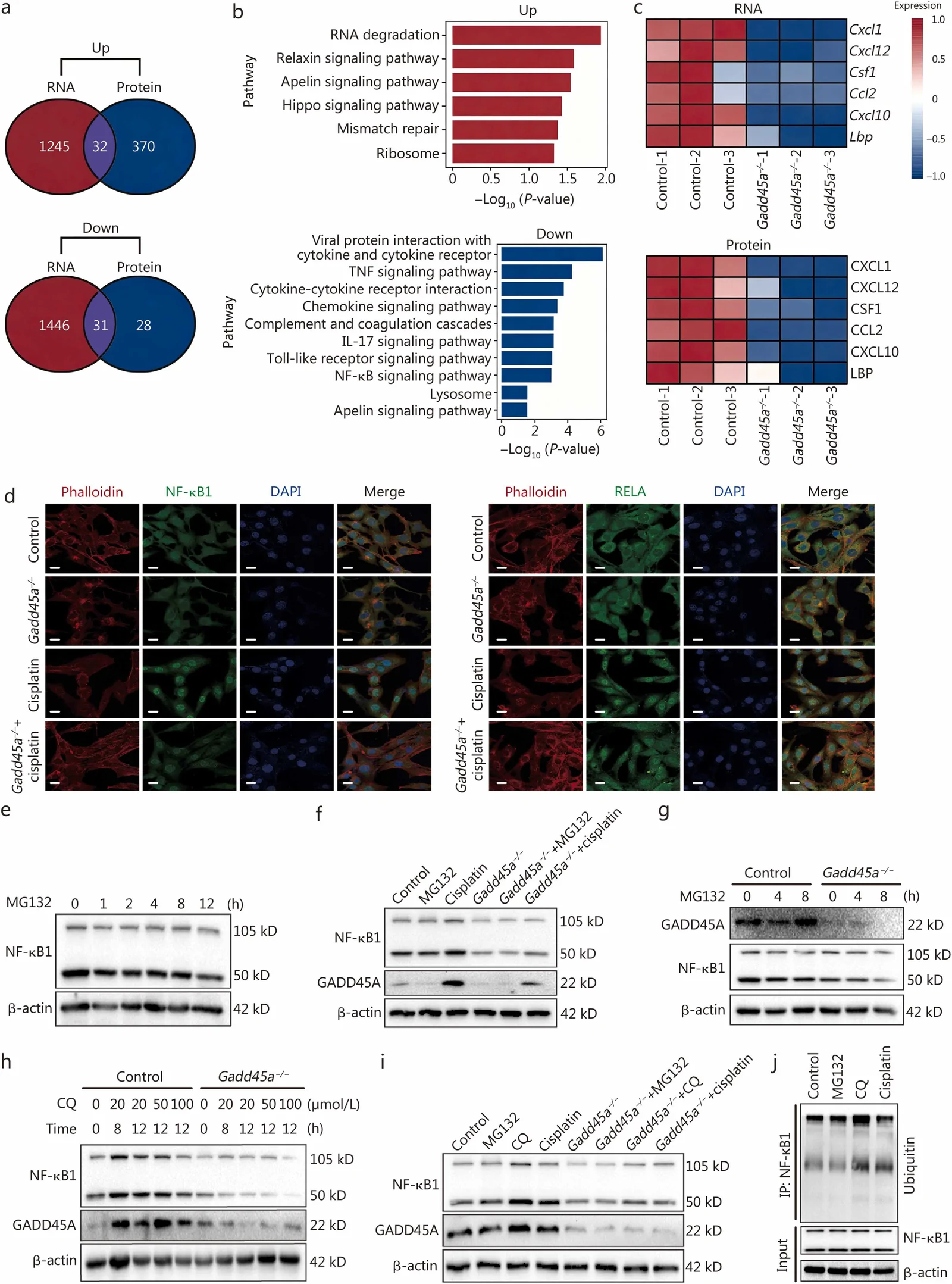
*Gadd45a* knockout affected NF-κB1 localization and its degradation by the lysosomal pathway. a Co-upregulated and -downregulated genes identified by RNA-protein intersection analysis. b KEGG pathway enrichment analysis of co- upregulated and downregulated genes identified by transcriptome-proteome intersection analysis. c Heatmaps showing the expression profiles of downregulated inflammatory cytokines at the transcriptomic (upper panel) and proteomic (lower panel) levels. d Representative image of NF-κB1 and RELA staining of control and *Gadd45a* knockout cell lines after treatment with 25 μmol/L cisplatin for 24 h. Scale bar=20 μm. e NF-κB1 protein expression after 10 μmol/L MG132 treatment for 1, 2, 4, 8, and 12 h. f NF-κB1 protein expression after 10 μmol/L MG132 treatment and 25 μmol/L cisplatin treatment. g NF-κB1 protein expression after 10 μmol/L MG132 treatment for 4 h and 8 h. h NF-κB1 protein expression in control and *Gadd45a* knockout groups treated with CQ at different concentrations and different time points. i NF-κB1 protein expression after 10 μmol/L MG132 treatment, 20 μmol/L CQ, and 25 μmol/L cisplatin treatment. j Protein expression of ubiquitination levels of NF-κB1 after treatment with MG132, CQ, and cisplatin. NF-κB1. Nuclear factor kappa B subunit 1; RELA. RELA proto-oncogene, NF-κB subunit; CXCL. C-X-C motif chemokine ligand; RNA. Ribonucleic acid; KEGG. Kyoto Encyclopedia of Genes and Genomes; GO. Gene Ontology; CQ. Chloroquine; MG132. Carbobenzoxy-Leu-Leu-leucinal; IP. Immunoprecipitation.

To integrate the transcriptomic and proteomic datasets, we performed RNA-protein intersection analysis. The analysis revealed intersecting elements, 32 of which were upregulated and 31 of which were downregulated (Fig. 4**a**). KEGG pathway analysis revealed that the intersecting downregulated pathways were predominantly associated with inflammatory and immune responses, including chemokine signaling, cytokine-cytokine receptor interaction, TNF signaling, IL-17 signaling, and NF-κB signaling (Fig. 4**b**). In contrast, the intersecting upregulated pathways were mainly involved in RNA metabolism and repair-related processes, such as RNA degradation and mismatch repair. Among the downregulated overlapping molecules, CXCL1, CXCL12, colony stimulating factor 1 (CSF1), C-C motif chemokine ligand 2 (CCL2), CXCL10, and lipopolysaccharide-binding protein (LBP) exhibited consistent decreases at both the transcriptomic and proteomic levels (Fig. 4**c**). Immunofluorescence staining further demonstrated that *Gadd45a* knockout attenuated cisplatin-induced NF-κB1 nuclear translocation, whereas RELA localization remained largely unchanged (Fig. 4**d**). Together, these findings suggest that GADD45A regulates inflammatory signaling through NF-κB1-dependent mechanisms.

### 3.4 GADD45A maintains NF-κB1 stability by preventing its lysosomal degradation

The process of ubiquitination, a type of post-translational modification involving the covalent binding of ubiquitin molecules to specific target proteins, is essential for maintaining cellular homeostasis. MG132, a well-characterized proteasome inhibitor, directly suppresses proteasomal activity, thereby preventing the degradation of ubiquitinated proteins. NF-κB1 protein levels did not accumulate following treatment with 10 µmol/L MG132 for the indicated durations (Fig. 4**e;** Additional file 1**:** Fig. S4a), suggesting that NF-κB1 may not be a substrate for proteasomal degradation. To further determine whether the reduction in NF-κB1 induced by *Gadd45a* deficiency could be rescued by proteasome inhibition, MG132 was applied for 12 h to control and *Gadd45a*^−/−^ cells (Fig. 4**f;** Additional file 1**:** Fig. S4b). MG132 treatment failed to increase NF-κB1 expression in either control or *Gadd45a*^−/−^ cells. To assess whether NF-κB1 protein levels were altered at additional MG132 treatment time points, NF-κB1 expression was examined after MG132 exposure for the indicated durations (Fig. 4**g;** Additional file 1**:** Fig. S4c). NF-κB1 expression remained largely unchanged, further supporting that proteasome inhibition had limited effects on NF-κB1 protein stability under these experimental conditions.

In addition to proteasomal degradation, lysosomal degradation represents another major pathway. Chloroquine (CQ), a classical lysosomal inhibitor, exerts its effects by increasing intra-lysosomal pH, thereby inhibiting the activity of lysosomal hydrolases. CQ treatment resulted in the accumulation of NF-κB1 protein in control cells and was not observed in the *Gadd45a* knockout group (Fig. 4**h;** Additional file 1**:** Fig. S4d). These findings suggest that NF-κB1 is more likely to be degraded through the lysosomal pathway than through the proteasome pathway. We next compared the effects of MG132 and CQ in control and *Gadd45a*^−/−^ cells. CQ treatment, but not MG132 treatment, resulted in marked accumulation of NF-κB1 protein in control cells (Fig. 4**i;** Additional file 1**:** Fig. S4e). Co-immunoprecipitation (co-IP) analysis showed that CQ treatment increased the ubiquitin signal associated with NF-κB1, whereas MG132 treatment had little effect, and revealed ubiquitinated NF-κB1, with increased ubiquitin signals following CQ treatment and cisplatin treatment (Fig. 4**j;** Additional file 1**:** Fig. S4f). Combined with the results of MG132 and CQ treatment, these findings indicate that ubiquitinated NF-κB1 is primarily degraded through the lysosomal pathway rather than the proteasomal pathway.

Although Flag*-Gadd45a* expression was detectable after transfection, it progressively decreased during three consecutive passages and was nearly undetectable by passage 3, preventing the establishment of a stable *Gadd45a*-overexpressing cell line (Additional file 1**:** Fig. S4g**-h**). Transient overexpression followed by co-IP assays did not detect a stable association of GADD45A with NF-κB1, RELA, or phosphorylated RELA (Additional file 1**:** Fig. S4j), suggesting the regulation of NF-κB1 by GADD45A may not involve stable direct binding.

### 3.5 Injection of si*Gadd45a* through the posterior semicircular canal reduces cisplatin-induced ototoxicity

Given the protective effects of *Gadd45a* deficiency observed *in vitro*, we next evaluated whether siRNA-mediated *Gadd45a* knockdown could alleviate cisplatin-induced ototoxicity *in vivo*. Accordingly, *Gadd45a*-targeting siRNA was delivered into the mouse inner ear through the PSC. The procedure for PSC injection is shown in Additional file 1**:** Fig. S5a. Following injection of 5’-FAM-labeled siRNA, strong fluorescence signals were detected in the dissected inner ear specimen, confirming successful delivery of siRNA into the inner ear. *In vivo* fluorescence imaging demonstrated persistent retention of 5’-FAM-siRNA in the injected ear region for up to 7 d after administration (Additional file 1**:** Fig. S5a).

To further improve the efficacy of cochlear *Gadd45a* expression suppression, we used Invivofectamine 3.0 Reagent, a non-animal-derived lipid nanoparticle. This reagent encapsulates siRNA for *in vivo* delivery, facilitating efficient uptake by living mouse cells without eliciting toxicity or stress responses. Western blotting (Additional file 1**:** Fig. S5b) and immunofluorescence staining (Additional file 1**:** Fig. S5c) confirmed that siRNA treatment significantly reduced *Gadd45a* expression in the cochlea.

**Fig. 5:**
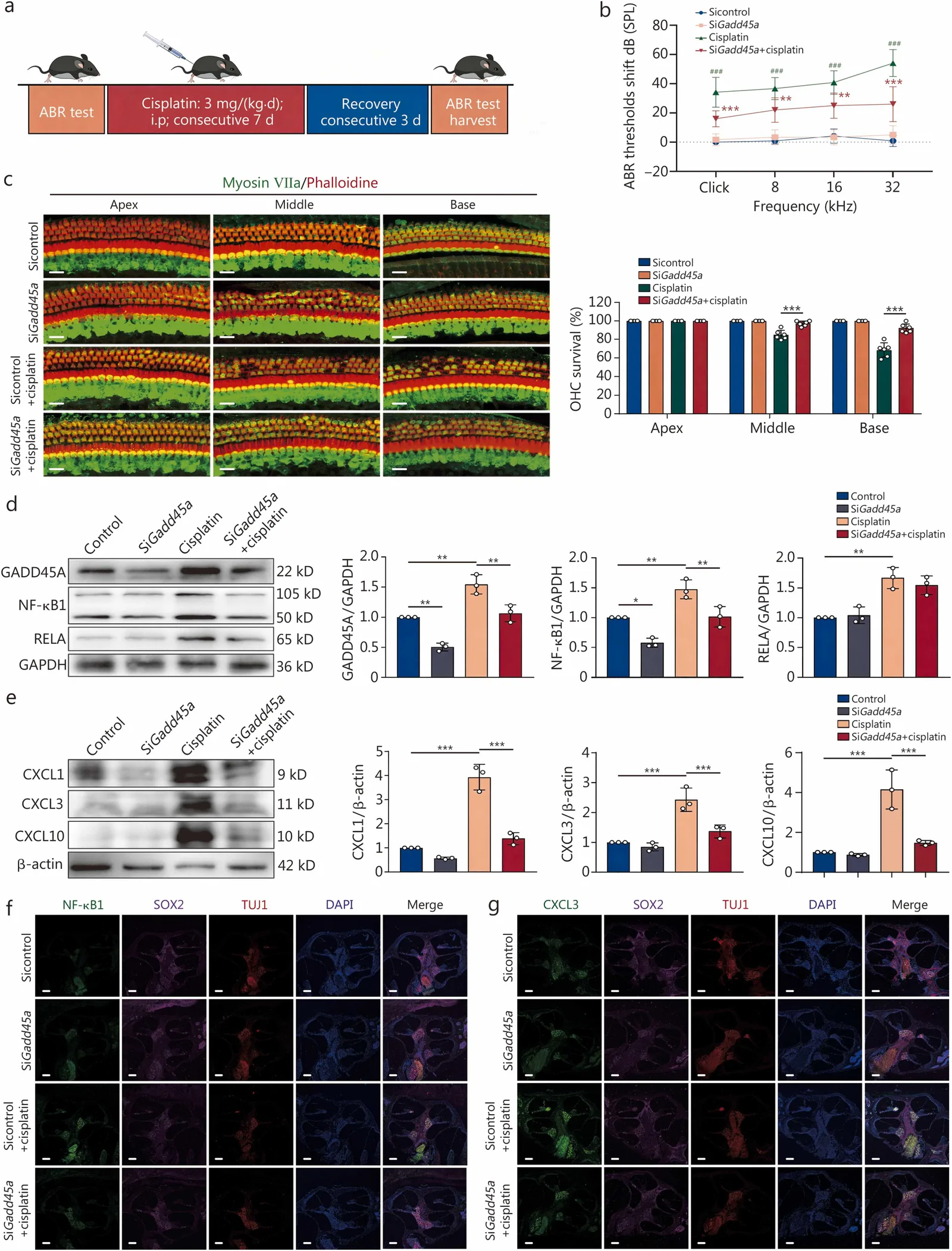
Injection of si*Gadd45a* through the PSC reduces cisplatin-induced ototoxicity. a Single cycle cisplatin injection pattern in mice. b ABR threshold shift results before and after cisplatin injection. ^**^P<0.01, ^***^P<0.001 vs. cisplatin group; ^###^*P*<0.001 vs. sicontrol group. c Representative immunofluorescence images and quantification of outer hair cell (OHC) survival following cisplatin administration. Scale bar=20 μm. d Representative Western blotting images and quantification of GADD45A, NF-κB1, and RELA expression in cochlear tissues following cisplatin administration. e Representative Western blotting images and quantification of CXCL1, CXCL3, and CXCL10 expression in cochlear tissues from control and si*Gadd45a*-treated mice following cisplatin administration. f Immunofluorescence staining of NF-κB1 in the cochlea. Scale bar=100 μm. g Immunofluorescence staining of CXCL3 in the cochlea. Scale bar=100 μm. ^∗^*P*<0.05, ^∗∗^*P*<0.01, ^∗∗∗^*P*<0.001; horizontal bars indicate the groups compared. ABR. Auditory brainstem response; SPL. Sound pressure level; i.p. Intraperitoneal injection; NF-κB1. Nuclear factor κB subunit 1; RELA. RELA proto-oncogene, NF-κB subunit; CXCL. C-X-C motif chemokine ligand; GAPDH. Glyceraldehyde-3-phosphate dehydrogenase; SOX2. SRY-box transcription factor 2; TUJ1. Class III beta-tubulin; DAPI. 4’,6-diamidino-2-phenylindole.

To evaluate the efficacy of si*Gadd45a* knockdown in mitigating cisplatin ototoxicity, we first performed audiometry on mice to establish a baseline for subsequent experiments. Mice with qualified baseline audiometric profiles were selected for si*Gadd45a* injection via the PSC. On the second day, the mice were intraperitoneally injected with cisplatin. Subsequent audiometry was performed (Fig. 5**a**), followed by euthanasia and cochlear basement membrane immunofluorescence staining. si*Gadd45a* treatment significantly reduced cisplatin-induced ABR threshold shifts and preserved OHCs, particularly in the middle and basal turns of the cochlea (Fig. 5**b, c**). To investigate whether si*Gadd45a* injected into the cochlea could alleviate cisplatin-induced ototoxicity by modulating the inflammatory signaling pathway, we examined the expression of proteins associated with the inflammatory signaling pathway. Our results demonstrated that si*Gadd45a* effectively reduced the expression of the transcription factors NF-κB1(Fig. 5**d**), which subsequently led to the downregulation of the expression of the inflammatory cytokines CXCL1, CXCL3, and CXCL10 (Fig. 5**e**). Furthermore, immunofluorescence staining showed increased NF-κB1 and CXCL3 expression in the cochleae of cisplatin-treated mice, whereas this increase was reduced following si*Gadd45a* administration (Fig. 5**f, g)**. GADD45A immunostaining showed markedly reduced fluorescence intensity in the cochlea following si*Gadd45a* treatment. The knockdown efficiency of si*Gadd45a* in the cochlea was confirmed by GADD45A immunostaining (Additional file 1**:** Fig. S5d).

### 3.6 *Gadd45a* knockout alleviates cisplatin-induced ototoxicity *in vivo*

**Fig. 6:**
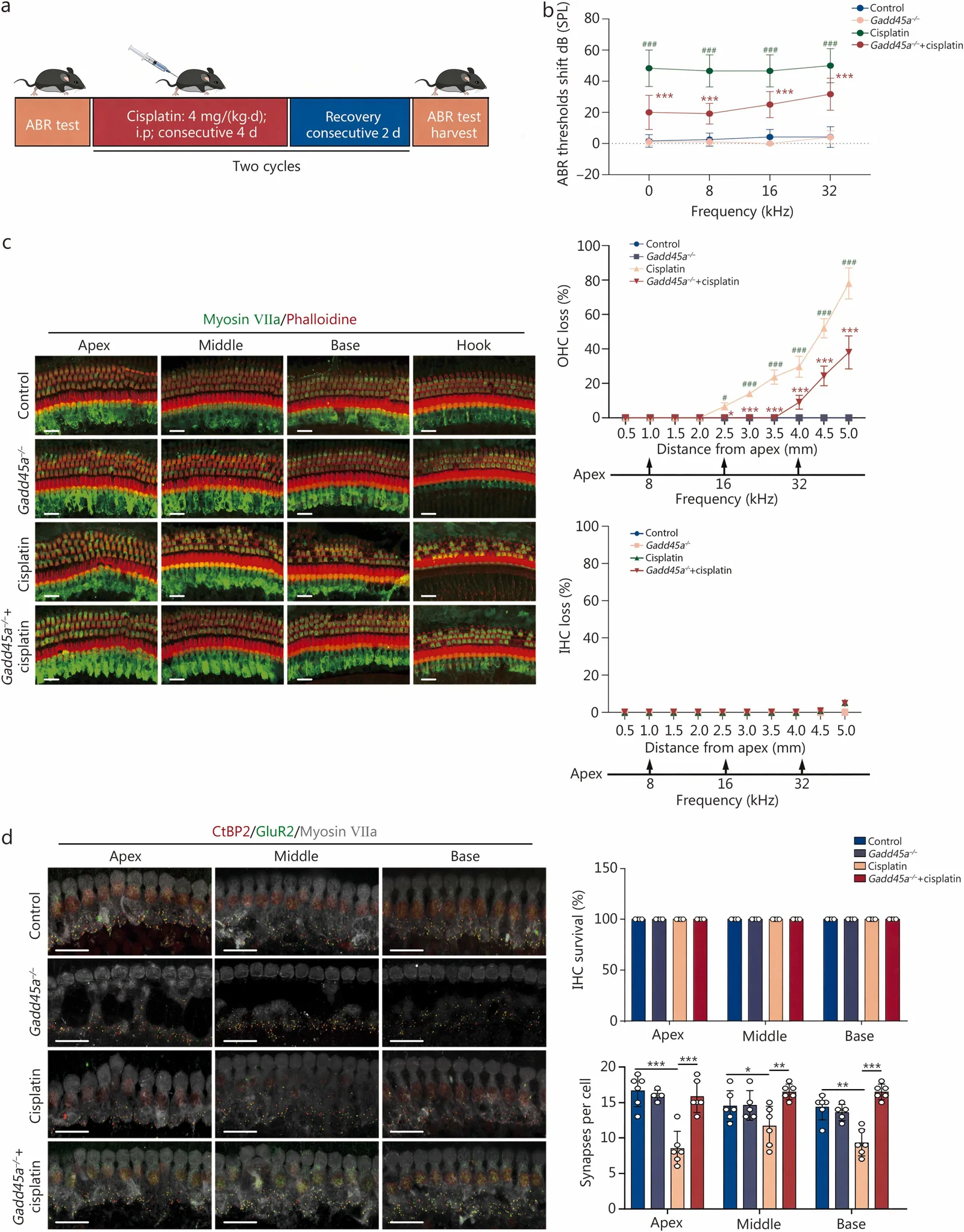
*Gadd45a* knockout can alleviate cisplatin ototoxicity. a Double cycle cisplatin intraperitoneal injection pattern in mice. b ABR test results of baseline levels of hearing and after cisplatin injection. c Representative immunofluorescence images of cochlear hair cells and quantification of outer hair cell (OHC) and inner hair cell (IHC) survival following cisplatin administration. Scale bar=20 μm. d Representative images of synaptic immunofluorescence staining and quantification of IHC survival and paired synapses per IHC. Scale bar=20 μm. *P<0.05, **P<0.01, ***P<0.001 vs. cisplatin group; ^#^*P*<0.05, ^###^*P*<0.001 vs. control group (b, c); *P<0.05^, *^*P<0.01, ***P<0.001 (d). ABR. Auditory brainstem response; SPL. Sound pressure level; i.p. Intraperitoneal injection; OHC. Outer hair cell; IHC. Inner hair cell; CtBP2. C-terminal binding protein 2; GluR2. Glutamate receptor 2; GADD45A. Growth arrest and DNA damage-inducible alpha; *Gadd45a*^−/−^. *Gadd45a* knockout.

A *Gadd45a* knockout allele was generated by inserting LoxP sites flanking exons 1–3. Cre-mediated recombination excises this region and disrupts the *Gadd45a* coding sequence (Additional file 1**:** Fig. S6a). *Gadd45a* cKO mice underwent two cycles of cisplatin treatment, with each cycle consisting of 4 consecutive days of cisplatin administration followed by a 2-day recovery period (Fig. 6**a**). Notably, compared with saline-treated mice, cisplatin-treated mice presented a substantial degree of body weight loss, regardless of whether they were conditional gene knockout mice (Additional file 1**:** Fig. S6b). We utilized immunofluorescence staining of cochlear cryosections to assess the efficiency of *Gadd45a* knockout. Since the GADD45A protein is predominantly localized to the nucleus, the absence of GADD45A in the nuclei of hair cells following *Gadd45a* knockout was evident. In contrast, GADD45A expression remained intact in the nuclei of supporting cells expressing SRY-box transcription factor 2 (SOX2) (Additional file 1**:** Fig. S6c). To determine whether *Gadd45a* knockout affects cisplatin-induced hearing loss, ABRs were measured in both control and *Gadd45a*^-/-^ mice. Baseline auditory function was determined by conducting auditory tests before cisplatin pretreatment. After the second cycle of cisplatin injection, ABR threshold shifts were significantly lower in cisplatin-treated *Gadd45a* knockout mice than in cisplatin-treated mice (Fig. 6**b**). Representative ABR waveform images at 32 kHz are shown in Additional file 1**:** Fig. S6d.

The mice were sacrificed at the end of the hearing test, and the inner ear tissues were collected for immunofluorescence staining (Fig. 6**c**). The whole cochlea was stained with Myosin VIIa (green) and phalloidin (red) to observe hair cell integrity. In control and *Gadd45a* knockout mice without cisplatin treatment, the cochlear morphology was normal, with 3 rows of OHCs and 1 row of IHCs aligned and no hair cell loss. Compared with cisplatin mice, *Gadd45a* knockout mice showed increased OHC survival after cisplatin treatment (Fig. 6**c**). Notably, Cochlear mapping showed that cisplatin induced substantial OHC loss in control mice, with the greatest loss occurring in the middle-to-basal region, whereas *Gadd45a* knockout significantly reduced OHC loss at these locations. In contrast, IHC loss remained minimal throughout the cochlea in both cisplatin-treated control and *Gadd45a*^−/−^ groups (Fig. 6**c**). Previous literature has shown that although IHCs remain intact after cisplatin treatment, there is a significant loss of specialized ribbon synapses [49]. To evaluate the impact of cisplatin on IHC synapses, whole-mount cochlear sections were stained for CtBP2 to label presynaptic ribbons and GluR2, an AMPA receptor subunit, to label postsynaptic glutamate receptors. The number of colocalized CtBP2 and GluR2 synapses per IHC was quantified and statistically analyzed. Significant synaptic loss was observed in cisplatin mice across all cochlear regions, whereas this reduction was not observed in cisplatin-treated *Gadd45a* knockout mice (Fig. 6**d**).

Cisplatin causes damage to the density of SGNs at the apex, whereas no such damage has been observed in the middle or basal regions of the cochlea [50]. To explore the impact of cisplatin on SGNs, cochlear sections were subjected to hematoxylin-eosin (H&E) staining. In our study, quantitative analyses revealed that the SGN density in the apex, middle, or base of the cochlea was not affected by cisplatin. Consistent with the hair cell survival analysis, H&E staining revealed more pronounced morphological alterations in hair cells than in SGNs or the stria vascularis following cisplatin treatment (Additional file 1**:** Fig. S6e). Additionally, the thickness of the stria vascularis was measured after cisplatin treatment, revealing that the stria vascularis was damaged, likely due to its role as the primary route for cisplatin entry (Additional file 1**:** Fig. S6e).

## 4 Discussion

**Fig. 7:**
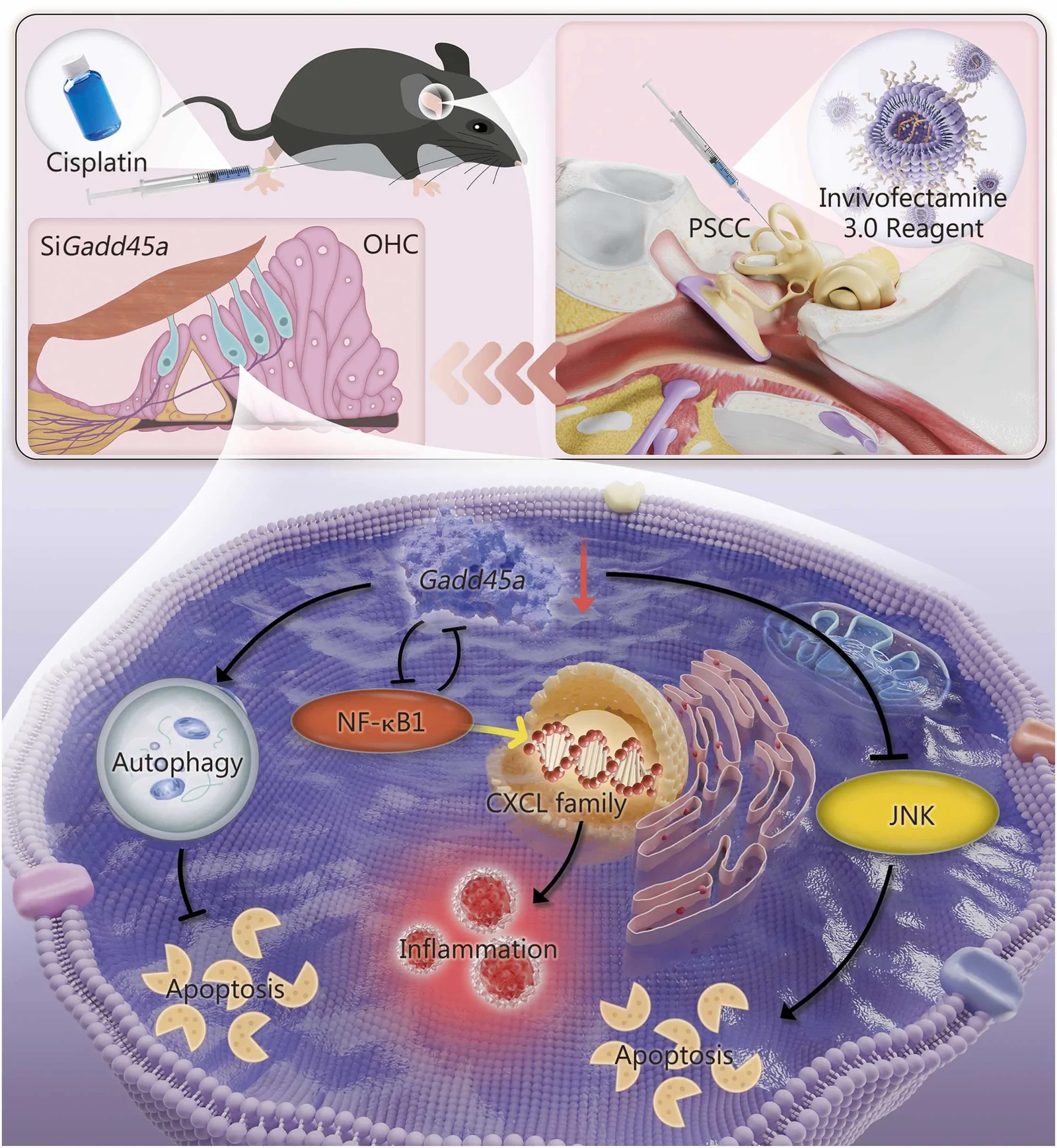
Schematic representation of the mechanism of si*Gadd45a*-mediated protection of hair cells from cisplatin damage. Cisplatin treatment induces Gadd45a expression, which promotes NF-κB1 activation and CXCL family-mediated inflammatory responses, leading to hair cell apoptosis. Silencing of GADD45A suppresses NF-κB1/CXCL signaling and attenuates inflammation and apoptosis, thereby protecting hair cells from cisplatin-induced injury. Si*Gadd45a*. Small interfering RNA targeting Gadd45a; OHC. Outer hair cell; GADD45A. Growth arrest and DNA damage-inducible alpha; NF-κB1. Nuclear factor kappa B subunit 1; CXCL. C-X-C motif chemokine ligand; JNK. c-Jun N-terminal kinase. PSCC. Posterior semicircular canal.

The present study identifies GADD45A as a critical mediator of cisplatin-induced ototoxicity. Through CRISPR-Cas9-mediated gene knockout, siRNA-mediated knockdown, cKO mouse models, we demonstrated that *Gadd45a* deficiency attenuated cisplatin-induced cochlear hair cell loss and hearing impairment. Mechanistically, *Gadd45a* deficiency reduced NF-κB1 protein levels, suppressed the expression of CXCL family chemokines, enhanced autophagic activity, attenuated apoptotic signaling, and ultimately preserved cochlear structural integrity and hearing function (Fig. 7). These findings suggest that GADD45A contributes to cisplatin-induced ototoxicity through coordinated regulation of stress responses, inflammation, and cell survival pathways.

The fluctuation of platinum tolerance-related gene expression during 24 h of cisplatin treatment indicates that modulating the expression of these genes may mitigate cisplatin-induced ototoxicity [14], [45], [48]. Although manipulation of *Top2a* [46], [48] and *Birc5* [45] did not produce significant protection in our experimental models, genetic ablation of *Gadd45a* markedly attenuated cisplatin-induced cochlear damage, identifying *Gadd45a* as a key regulator of cisplatin ototoxicity. Previous studies have primarily linked GADD45A to DNA damage responses and stress-induced apoptosis through activation of the MKK4-JNK signaling pathway [29], [51]. Consistent with these reports, *Gadd45a* expression was markedly induced following cisplatin exposure in our study. Notably, *Gadd45a* deficiency significantly reduced TUNEL^+^ cells, suggesting that GADD45A contributes to cisplatin-induced apoptotic signaling and subsequent cell death. These findings further extend the current understanding of GADD45A by the protective effect of *Gadd45a* deficiency in cochlear hair cells during cisplatin-induced ototoxicity.

Time-course cisplatin treatment experiments were subsequently performed to investigate the mechanisms underlying cisplatin tolerance. The temporal dynamics of the expression of DNA damage markers, particularly γ-H2A.X, revealed critical insights into the protective mechanism of *Gadd45a* knockout. These findings align with our observations that autophagic flux is enhanced, which may facilitate the clearance of cisplatin-DNA adducts through alternative repair mechanisms. The conserved nature of this response across tumor and cochlear cell types highlights the evolutionary importance of GADD45A-mediated stress adaptation. Previous studies have shown that activation of autophagy protects cochlear hair cells from cisplatin-induced damage by reducing oxidative stress and apoptosis [52], [53]. Our findings are consistent with these observations and suggest that enhanced autophagic flux may contribute to the protective phenotype observed in *Gadd45a*-deficient cells.

To further elucidate the mechanisms underlying GADD45A-mediated cisplatin tolerance, we conducted transcriptomic sequencing. Transcriptomic profiling revealed a striking downregulation of the expression of chemokine signaling components (CXCL1/3/10/12) in *Gadd45a*-deficient cells, implicating inflammatory regulation as a novel facet of *Gadd45a* biology. This finding was further supported by reduced NF-κB1 expression and decreased CXCL1/3/10 protein levels in both cultured cells and cochlear tissues, indicating that *Gadd45a* deficiency suppresses inflammatory signaling. Inflammatory signaling has been increasingly recognized as a key contributor to cisplatin-induced ototoxicity. Prior research has demonstrated that inhibiting CXCL1 effectively reduces cisplatin ototoxicity [25]. Moreover, the increase in inflammatory mediators, such as TNF-α, was attenuated by *Stat1* siRNA, and cells were protected from cisplatin-mediated apoptosis due to this inhibition [54]. The downregulation of NF-κB expression by flunarizine through the activation of the nuclear factor erythroid 2-related factor 2/heme oxygenase-1 (Nrf2/HO-1) pathway has been shown to reduce cisplatin-induced ototoxicity, thereby inhibiting the production of proinflammatory cytokines both *in vitro* and *in vivo* [55].

Previous research has shown that the homeostasis of the NF-κB family is intricately linked to the expression levels of inflammatory factors [56]. We identified NF-κB1 (p105/p50) and RELA (p65) as potential targets using transcription factor prediction and scoring software. We next explored the relationship between GADD45A and NF-κB. GADD45A may be regulated by NF-κB1, which mediates the activation of the GADD45A-MKK4-JNK cascade and apoptosis [29]. However, the role of this transcription factor in modulating GADD45A expression remains controversial. In contrast, our findings suggest that GADD45A can also regulate NF-κB1 stability and activity, indicating a potentially bidirectional interaction between these pathways. NF-κB1 expression decreased while RELA increased when *Gadd45a* was knocked out, demonstrating that this chemokine suppression occurs through NF-κB1-dependent mechanisms rather than through canonical RELA pathways. The differential regulation of NF-κB subunits suggests a previously unrecognized specificity in GADD45A-mediated inflammatory control, potentially explaining the broad-spectrum anti-inflammatory effects observed in the knockout models.

Since GADD45A is involved in the regulation of inflammatory factors, which are typically produced by macrophages, we investigated the cellular sources of cochlear inflammation. In the normal physiological state of the cochlea, resident macrophages are present in a dormant state within the cochlear tissue. Upon exposure to cisplatin, these resident macrophages become activated, resulting in the release of inflammatory mediators. This activation, in turn, stimulates peripheral immune cells to infiltrate damaged areas of the cochlea, thereby amplifying the inflammatory response [50]. OC1 is an immortalized cell line, originating from the cochlear sensory epithelium. Because CXCL1, CXCL3, and CXCL10 are secreted proteins, BFA was used to inhibit protein secretion and promote their intracellular accumulation before cell lysis. We found that the CXCL1, CXCL3, and CXCL10 proteins were significantly expressed in OC1 cells, with no detectable signal in the *Gadd45a* knockout group. Immunofluorescence localization studies revealed impaired nuclear translocation of NF-κB1 in knockout cells, indicating that *Gadd45a* influences both the expression and activation dynamics of this transcription factor. This dual regulatory capacity positions GADD45A as a master coordinator of inflammatory responses to ototoxic stress [56].

Several limitations should be acknowledged. First, although our data support a role for GADD45A in regulating NF-κB1-dependent inflammatory signaling, the precise molecular mechanism by which GADD45A controls NF-κB1 lysosomal degradation requires further investigation. Second, although the molecular mechanisms were extensively investigated in OC1 cells, cell type-specific validation within the cochlea remains necessary. Third, although conditional *Gadd45a* knockout and siRNA-mediated knockdown both conferred protection against cisplatin ototoxicity, the long-term effects of *Gadd45a* inhibition on cochlear homeostasis and auditory function remain unknown. Future studies addressing these questions will be important for evaluating the therapeutic potential of targeting GADD45A in hearing loss.

## 5 Conclusion

In conclusion, this work identifies GADD45A as a multimodal regulator of cisplatin ototoxicity through three convergent mechanisms: 1) JNK-mediated apoptotic regulation, 2) autophagic pathway activation, and 3) NF-κB1-dependent inflammatory suppression. The clinical relevance of these findings is underscored by successful *in vivo* protection in *Gadd45a* knockout models, suggesting that the targeted inhibition of *Gadd45a* signaling could preserve auditory function during cisplatin chemotherapy. Future investigations should focus on temporally specific GADD45A expression modulation and advanced delivery systems to translate these findings into clinically viable otoprotective strategies.

## Abbreviations

ABR: Auditory brainstem response AKI: Acute kidney injury AMPK: AMP-activated protein kinase BAX: BCL2-associated X protein BCL-2: B-cell lymphoma 2 BECN1: Beclin 1 BFA: Brefeldin A BIRC5: Baculoviral IAP repeat containing 5 CCK-8: Cell Counting Kit-8 CCL2: C-C motif chemokine ligand 2 cKO: Conditional knockout co-IP: Co-immunoprecipitation CQ: Chloroquine CSF1: Colony stimulating factor 1 CtBP2: C-terminal binding protein 2 CXCL: C-X-C motif chemokine ligand CXCL1: C-X-C motif chemokine ligand 1 CXCL3: C-X-C motif chemokine ligand 3 CXCL10: C-X-C motif chemokine ligand 10 EDTA: Ethylenediaminetetraacetic acid disodium salt FAM: Fluorescein amidite FDA: U.S. Food and Drug Administration GADD45A: Growth arrest and DNA damage-inducible alpha GluR2: AMPA-type glutamate receptor subunit 2 GO: Gene Ontology GSEA: Gene set enrichment analysis HO-1: Heme oxygenase-1 IBA1: Ionized calcium-binding adaptor molecule 1 IHC: Inner hair cell IL-17: Interleukin-17 IPR: InterPro IκB: Inhibitor of nuclear factor κB JNK: c-Jun N-terminal kinase KEGG: Kyoto Encyclopedia of Genes and Genomes KOGs: Eukaryotic orthologous groups of proteins LBP: Lipopolysaccharide-binding protein LC3-II: Microtubule-associated protein 1 light chain 3-II LC3B-II: Microtubule-associated protein 1 light chain 3 beta-II LPS: Lipopolysaccharide MKK4: Mitogen-activated protein kinase kinase 4 NF-κB: Nuclear factor κB NF-κB1: Nuclear factor κB subunit 1 Nrf2: Nuclear factor erythroid 2-related factor 2 OHC: Outer hair cell PBS: Phosphate-buffered saline PCR: Polymerase chain reaction PFA: Paraformaldehyde PI: Propidium iodide PIK3C3: Phosphatidylinositol 3-kinase catalytic subunit type 3 PSC: Posterior semicircular canal RELA: RELA proto-oncogene, NF-κB subunit ROS: Reactive oxygen species SGN: Spiral ganglion neuron

SV:Strial Vascularis SOX2: SRY-box transcription factor 2 SPL: Sound pressure level SQSTM1: Sequestosome 1 TDT: Tucker-Davis Technologies TNF: Tumor necrosis factor TNF-α: Tumor necrosis factor alpha TOM20: Translocase of outer mitochondrial membrane 20 homolog TOP2A: DNA topoisomerase II alpha γ-H2A.X: Phosphorylated H2A histone family member X

## Ethics approval and consent to participate

All animal experiments were approved and supervised by the Laboratory Animal Welfare & Ethics Committee of Zhongnan Hospital of Wuhan University (WP20240254). The procedures of the animal experiments complied with the National Institutes of Health Guide for the Care and Use of Laboratory Animals.

## Authors’ contributions

ZHH conceptualized the study, secured funding, supervised the project, and provided final manuscript editing. WLW played a central role in conducting the experiments, analyzing the datasets, and initially drafting the manuscript. SYZ performed the semicircular canal microinjection experiment and collected cochlear tissue samples. DQW and YL performed immunofluorescence staining and mouse genotyping. JWL, FZK, and SHW were responsible for the analysis of the bioinformatics data. BWX drew the graphical abstract. KL and CJW procured laboratory reagents and consumables. WLW, XXX, XC, XLF, QJF, and ZHH were involved in discussions about the experimental protocol and data interpretation. All the authors have read and approved the final manuscript.

## Funding

This work was supported by the National Key Research and Development Program of China (2023YFA1801801 and 2024YFA1108200), the National Natural Science Foundation of China (82222017, 82271183, 82571327, 82301319, and 82271181), the Jiangsu Provincial Key Science and Technology Program (BG2024037), and the Hubei Provincial Key Health Science and Technology Program (WJ2025Z006).

## Data Availability

All data are available in the main text or the Additional file 1. The datasets generated and analyzed during the current study are available in public repositories. The raw RNA-seq data are available in the Genome Sequence Archive under accession number PRJCA054899. The mass spectrometry proteomics raw data are available in the iProX database under accession number PXD072616. The datasets generated and analyzed during the current study are available from the corresponding author upon reasonable request.

## Footnotes

Appendix A Supplementary data associated with this article can be found in the online version at doi:10.1016/j.mmr.2026.100063.
