# Supplementary material for: Gadd45a knockout alleviates cisplatin-induced hearing loss by inhibiting CXCL family protein expression
Source: Mil Med Res. 2026 Aug 14;13(1):100063. doi: 10.1016/j.mmr.2026.100063 (PMC13499120; doi:10.1016/j.mmr.2026.100063)
Supplement: Supplementary file 1 [file mmc1.pdf]

Methods

Genotype identification

Genomic DNA was extracted from a small tail or toe fragment from heterozygous mice using a 50 mmol/L NaOH solution at 95 °C for 1 h. Polymerase chain reaction (PCR) amplification was then used to identify the genotype. Primer sequences are shown in the **Additional file**: **Table S1**. The F1 mouse strain that was growth factor independent 1-Cre recombinase (*GFI*^{Cre}) positive was designated *GFI*$^{Cre+}$/growth arrest and DNA damage-inducible alpha (*Gadd45a*$^{-/-}$). These mice were then crossbred with *Gadd45a*$^{-/-}$ mice. The resulting F2 mouse strain, *GFI*$^{Cre+}$/*Gadd45a*$^{-/-}$, represents *Gadd45a* conditional knockout (cKO) mice. The genotyping results revealed that *GFI*$^{Cre-}$ mice presented a single band at 609 bp, whereas *GFI*$^{Cre+}$ mice presented two bands at 609 bp and 672 bp. For *Gadd45a* knockout mice, homozygotes presented a single band at 318 bp, heterozygotes presented two bands at 318 bp and 250 bp, and wild-type alleles presented a single band at 250 bp.

Real-time PCR

Total RNA was extracted via TRIzol reagent (R411-01; Vazyme, Nanjing, China) following the standard procedure. The purified RNA was subsequently reverse transcribed into complementary DNA (cDNA) using 5× PrimeScript RT Master Mix for qPCR (RR047Q; TaKaRa, Japan). Quantitative RT-PCR was conducted on a StepOnePlus Real-Time PCR System (Applied Biosystems, USA) with TB Green Premix Ex Taq 2 (Tli RNaseH Plus, RR820Q, TaKaRa, Japan). *β-actin* was used as a housekeeping gene for normalization, and the relative expression levels of each gene were calculated using the $2^{-\Delta\Delta Ct}$ method. Primer sequences are exhibited in **Additional file 1**: **Table S3**.

Western blotting

OC1 and *Gadd45a* knockout cells were collected and lysed in RIPA buffer (P0013B; Beyotime Biotech Inc., China) containing a protease inhibitor cocktail (04693159001; Roche, Switzerland) for 30 min on ice with constant agitation. After centrifugation at 12,000 rpm (4 °C, 10 min), the supernatant was collected, and the total protein concentration was quantified using a BCA assay kit (P0012S; Beyotime Biotechnology Inc., China). The samples were then mixed with 5× SDS-PAGE loading buffer (P0015L; Beyotime Biotechnology Inc., China) and denatured by heating at 95 °C for 10 min.

The cell culture supernatants were collected and processed in batches using ultrafiltration concentration tubes (UFC9003; Merck Millipore, Billerica, USA). The samples were subsequently centrifuged at 4000 rpm for 15 min, and the centrifugation process was repeated three times. When the volume of the concentrated solution reached 500 μl, the solution was transferred to a 1.5 ml EP tube. The samples were then mixed with 5× SDS-PAGE loading buffer (P0015L; Beyotime Biotechnology Inc., China) and denatured by heating at 95 °C for 10 min.

For electrophoresis, 20 μg protein per lane was resolved via SDS-PAGE and subsequently transferred to PVDF membranes (ISEQ00010; Merck Millipore, Billerica, USA). Blocking was performed with 5% nonfat milk for 1 h at room temperature, followed by overnight incubation with primary antibodies at 4 °C under gentle shaking. The next day, the membranes were washed four times with TBST (10 min per wash) and incubated with HRP-conjugated secondary antibodies (Cell Signaling Technology, USA; 1:4000) for 1 h at room temperature with agitation. Finally, protein bands were detected using a chemiluminescence substrate (34580; SuperSignal West Pico PLUS, Thermo Fisher Scientific, USA) and imaged via a chemiluminescence system. Primary antibodies used for immunoblotting are listed in **Additional file 1**: **Table S4**.

Cell viability

A CCK-8 kit (A311-02-AA; Vazyme, China) was used to evaluate cell viability. On the first day, OC1 and *Gadd45a* cKO cells were individually plated in 96-well plates at a density of 5×10^3 cells per well and cultured overnight for cell adhesion. On the following day, the culture medium was replaced with fresh medium containing different concentrations of cisplatin, and the cells were treated for 24 h. After the model of cisplatin damage was established, 10 μl CCK-8 reaction mixture was combined with 90 μl fetal bovine serum-free DMEM and added to each well. The 96-well plates were then incubated at 37 °C for 1–2 h. The absorbance was measured at 450 nm using a multifunctional enzyme-labeled instrument (HH3500; PerkinElmer, USA).

Flow cytometry

An annexin V-FITC/ propidium iodide (PI) kit (556547; BD Biosciences Pharmingen, USA) was used to assess the proportion of apoptotic cells when the cells were exposed to cisplatin. On the first day, OC1 and *Gadd45a* knockout cells were separately seeded in 6-well plates at a density of 60%. The next day, the culture medium was replaced with fresh medium containing 25 μmol/L cisplatin, and the mixture was incubated for 24 h. The cells were then washed twice with precooled phosphate-buffered

saline (PBS), digested with trypsin, collected by centrifugation at 1000 rpm for 5 min, and resuspended at a concentration of 1×10^6 cells/ml. Annexin V-FITC and PI were added to the cell suspensions, which were subsequently incubated in the dark at room temperature for 15 min. A Beckman flow cytometer was used for data acquisition, and FlowJo software was used for data processing.

TUNEL assay

A TUNEL assay was performed following the manufacturer's protocols for an in situ cell death detection kit (Roche, Indianapolis, IN, USA). On the first day, OC1 and *Gadd45a* knockout cells were seeded in confocal dishes and treated with cisplatin. After the model was established, the cells were washed with precooled PBS and fixed with paraformaldehyde (PFA) at room temperature for 15 min. The samples were then permeabilized with 0.2% Triton X-100 for 15 min. Subsequently, 100 µl TUNEL reaction mixture was added to each confocal dish and incubated at 37 °C for 60 min. After TUNEL staining, DAPI was added at a concentration of 1:500, and the samples were incubated at room temperature for 10 min. Finally, a Leica confocal laser scanning microscope (Leica TCS SP8, Leica Microsystems GmbH, Wetzlar, Germany) was used to obtain images.

ELISA

The supernatants of the OC1 and *Gadd45a* knockout cells were concentrated using ultrafiltration devices (UFC9003; Millipore, USA) and then analyzed according to the protocols of a Rat CXC Chemokine Ligand-1 (CXCL1/IL-8) ELISA Assay Kit (Nanjing Jiancheng Bioengineering Institute, China).

Posterior semicircular canal injection strategy

The animals were deeply anesthetized via an intraperitoneal injection of ketamine (100 mg/kg, i.p.) and xylazine (25 mg/kg, i.p.). The skin behind the ear was prepared by clipping the hair and applying depilatory cream. The mouse was then positioned on an operating table (Yuyan Instruments, China), and its body temperature was maintained on an electric heating pad. A 1-cm postauricular incision was made behind the ear, and the sternocleidomastoid muscle was bluntly dissected via ophthalmic scissors. The facial nerve and bulla were identified, and the posterior semicircular canal (PSC) was located superiorly and posteriorly from the facial nerve. The muscle fibers and soft tissues overlying the PSC were removed, and the lumen of the PSC was accessed by drilling with a 27-gauge needle. Lymphatic leakage indicated successful access to the PSC lumen. A microinjection Syring Pump (249-1392;

World Precision Instruments, USA) was used to deliver the injection through a microliter syringe (φ0.31 mm, 10 μl, Shanghai Bolige Industry & Trade Co., Ltd., China) connected to a thin tube (1549360; Teleflex, USA) with an inner/outer diameter of 0.0030/0.0040 inches. The tube was inserted into the PSC with forceps. The space between the inserted tube and the PSC was sealed with superficial fascia and tissue adhesive (1469SB; 3M, USA) to prevent lymphatic leakage. The siRNA liposome mixture was injected into the PSC at a rate of 95 μl/h. After injection, the PSC fenestration was closed with adjacent muscle, and the incision was sealed with a swaged needle (F402, Jinhuan Medical Products, China). The wound was disinfected with an iodine wipe, and the mouse was placed on a heating pad for recovery.

Table S1 Primer sequences for genotyping

Gene	Primer
GFI-Cre-1	GCCCAAATGTTGCTGGATAGT
GFI-Cre-2	GGGATAACGGACCAGTTG
GFI-Cre-3	CCGAGGGGCGTTAGGATA
Gadd45a F1	TGCTGTCAAAGCTAAAGGTAGAAGT
Gadd45a F2	CGGTGAATTTGGCTCGTTAAACAT
Gadd45a R1	CCCTACACAAGTCAGAAGAGAAAGA

GFI. Growth factor independent 1 transcriptional repressor; Gadd45a. Growth arrest and DNA damage-inducible alpha

Table S2 Sequences of the sgRNAs

Gene	Primer
Top2a-1F	CACCGTTGACGAAGCTAATCTGC
Top2a-1R	AAACGCAGATTAGCTTCGTCAAC
Top2a-2F	CACCGAACAAGTAAATCCCCGGT
Top2a-2R	AAACACCGGGGATTTACTTGTTC
Birc5-1F	CACCGGAGGATTGCTCCTGCACC
Birc5-1R	AAACGGTGCAGGAGCAATCCTCC
Birc5-2F	CACCGACCGAGAATGAGCCTGAAT
Birc5-2R	AAACATTCAGGCTCATTCTCGGTC
Gadd45a-1F	CACCGTCAGCGCACCATAACTGTC
Gadd45a-1R	AAACGACAGTTATGGTGCGCTGAC
Gadd45a-2F	CACCGCGGGTCAGCAACCCGGGTC
Gadd45a-2R	AAACGACCCGGGTTGCTGACCCGC

Top2a. DNA topoisomerase II alpha; Birc5. Baculoviral inhibitor of apoptosis repeat-containing 5; Gadd45a. Growth arrest and DNA damage-inducible alpha

Table S3 Primer sequences for real-time PCR

Gene	Forward primer	Reverse primer
β-Actin	GTCCCTCACCCTCCCAAAAG	GTCCCTCACCCTCCCAAAAG
Gadd45a	GAGAACGACATCAACTCCTGC	CCACTGATCCATGTAGCGACTTT
Cxcl1	ACTGCACCCAAACCGAAGTC	AGCTTGAGGGTTGAGACAAACT
Cxcl3	TTTGAGAACATCCAGAGCTTGAC	AGCTTGAGGGTTGAGACAAACTT
Cxcl10	TATCCTGTCCGCATGTTGAGATC	TATCCTGTCCGCATGTTGAGATC
Cxcl12	TGCATCAGTGACGGTAAGCCA	CACAGTTTGGAGTGTTGAGGAT
Nf-κb1	GCGTCCAACCTGAAGATCGTAAG	ATCCTTCCCAAACTCCACCATTT
Rela	CTGTGTGACAAAGTGCAGAAAGA	GATCGTCTGTGTCTGGCAAGTA

β-actin. Beta-actin; Gadd45a. Growth arrest and DNA damage-inducible alpha; Cxcl. C-X-C motif chemokine ligand; Nf-kb1. Nuclear factor kappa B subunit 1; Rela. RELA proto-oncogene, NF-κB subunit

Table S4 Primary antibodies and chemical reagents

Antibody	Item No.	Company
TOP2A	ab52934	Abcam
BIRC5	ab76424	Abcam
GADD45A	#4632	CST
GADD45A	PA5-88248	Thermo
SQSTM1	ab10901	Abcam
LC3B	L7543	Sigma
BAX	#14796	CST
BCL-2	Ab182858	Abcam
C-CAS3	#9661s	CST
γ-H2A.X	ab81299	Abcam
AMPK	ab3759	Abcam
SOX2	14-9811-82	Thermo
CXCL1	ab86436	Abcam
CXCL3	ab220431	Abcam
CXCL10	ab9807	Abcam
Tubulin	80762-1-RR	ProteinTech
β-actin	20536-1-AP	ProteinTech
GAPDH	GTX100118	GeneTex
TOM20	11802-1-AP	ProteinTech
RELA (p65)	#4764	CST
p-RELA (p65)	#3033s	CST
NF-κB1(p50)	ab32360	Abcam
p-AMPK	#2531s	CST
TUJ1	ab78078	Abcam

TOP2A. DNA topoisomerase II alpha; BIRC5. Baculoviral inhibitor of apoptosis repeat-containing 5; GADD45A. Growth arrest and DNA damage-inducible alpha; SQSTM1. Sequestosome 1; LC3B. Microtubule-associated protein 1 light chain 3 beta; BAX. BCL2-associated X protein; BCL-2. B-cell lymphoma 2; C-CAS3. Cleaved caspase-3; γ-H2A.X. Phosphorylated histone H2A.X; AMPK. AMP-activated protein kinase; SOX2. SRY-box transcription factor 2; CXCL. C-X-C motif chemokine ligand; GAPDH. Glyceraldehyde-3-phosphate dehydrogenase; TOM20. Translocase of outer mitochondrial membrane 20; RELA.RELA proto-oncogene, NF-κB subunit; NF-κB1. Nuclear factor κB subunit 1; AMPK. AMP-activated protein kinase; TUJ1. Class III beta-tubulin

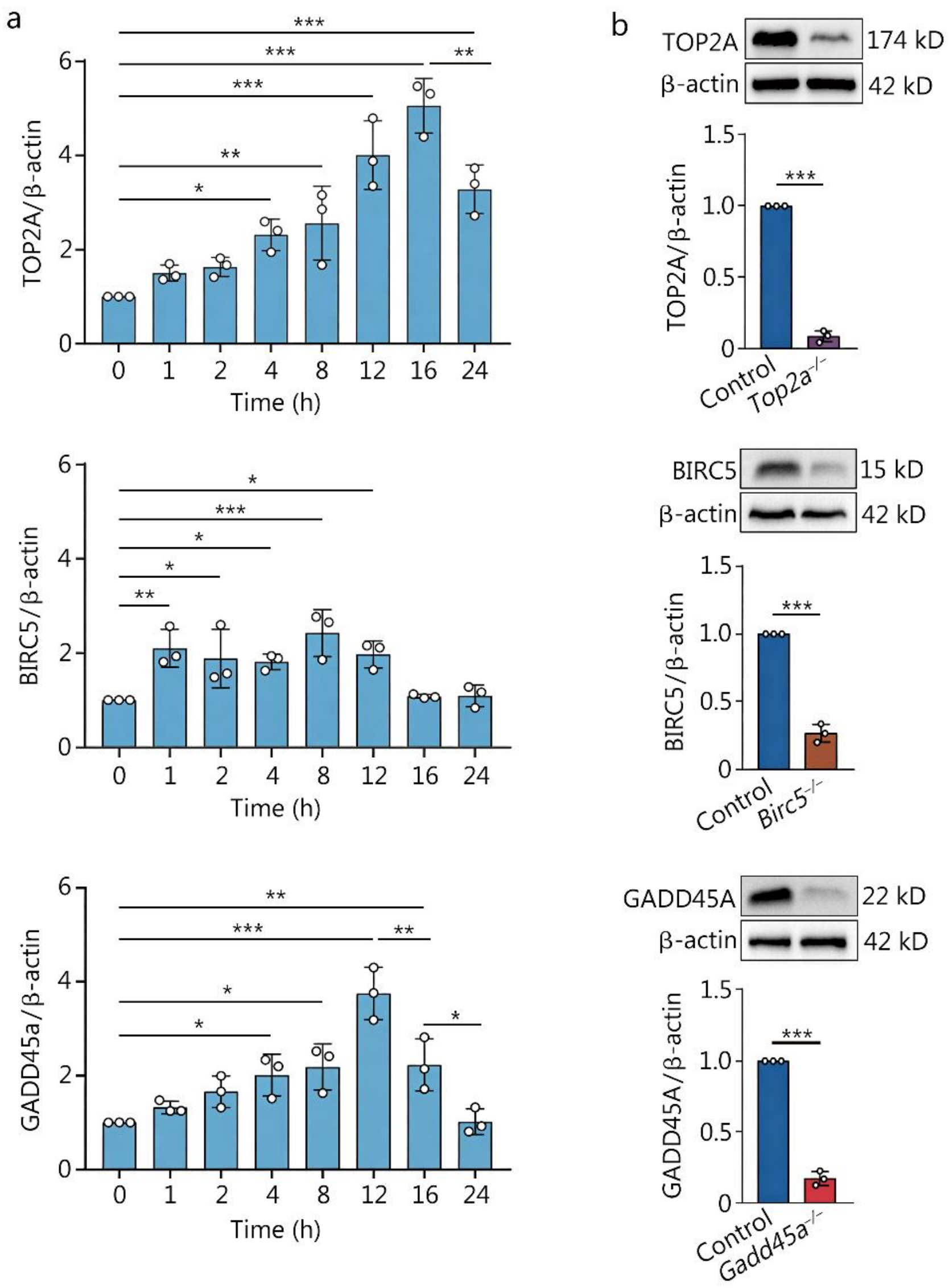

Fig. S1 Cisplatin-induced expression changes of TOP2A, BIRC5, and GADD45A and validation of knockout efficiency in OC1 cells. **a** Quantification of TOP2A, BIRC5, and GADD45A protein expression in OC1 cells treated with 25 μmol/L cisplatin for the indicated time points. β-actin was used as the loading control. **b** The knockout efficiency of *Top2a, Birc5,* and *Gadd45a* in OC1 cells and statistical analysis respectively. $^{*}P<0.05$, $^{**}P<0.01$, $^{***}P<0.001$; horizontal bars indicate the groups compared. TOP2A. DNA topoisomerase II alpha; BIRC5. Baculoviral inhibitor of apoptosis repeat-containing 5; GADD45A. Growth arrest and DNA damage-inducible alpha

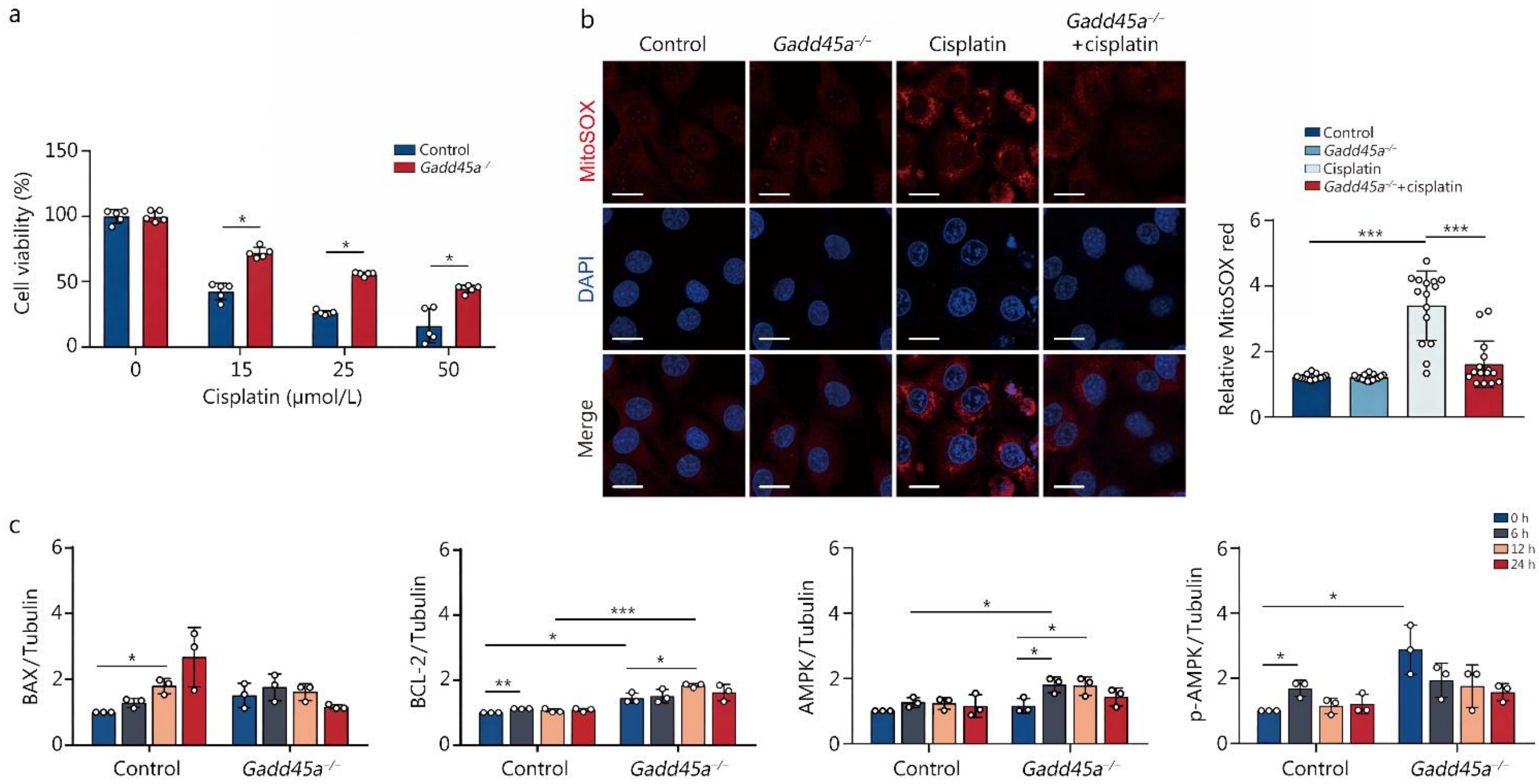

Fig. S2 *Gadd45a* knockout protects OC-1 cells from cisplatin-induced mitochondrial oxidative stress and apoptosis-related injury. **a** Cell viability of control and *Gadd45a*$^{-/-}$ OC1 cells treated with the different concentrations of cisplatin. **b** Representative fluorescence images and quantification of mitochondrial ROS levels in control and *Gadd45a*$^{-/-}$ OC1 cells with or without cisplatin treatment. Nuclei were counterstained with DAPI. Scale bar=20 μm. **c** Quantification of BAX, BCL-2, AMPK, and p-AMPK protein levels in control and *Gadd45a*$^{-/-}$ OC1 cells after cisplatin treatment for the indicated time points. $^{*}P<0.05$, $^{**}P<0.01$, $^{***}P<0.001$; horizontal bars indicate the groups compared. MitoSOX. Mitochondrial superoxide indicator; DAPI. 4',6-diamidino-2-phenylindole; BAX. BCL2-associated X protein; BCL-2. B-cell lymphoma 2; AMPK. AMP-activated protein kinase

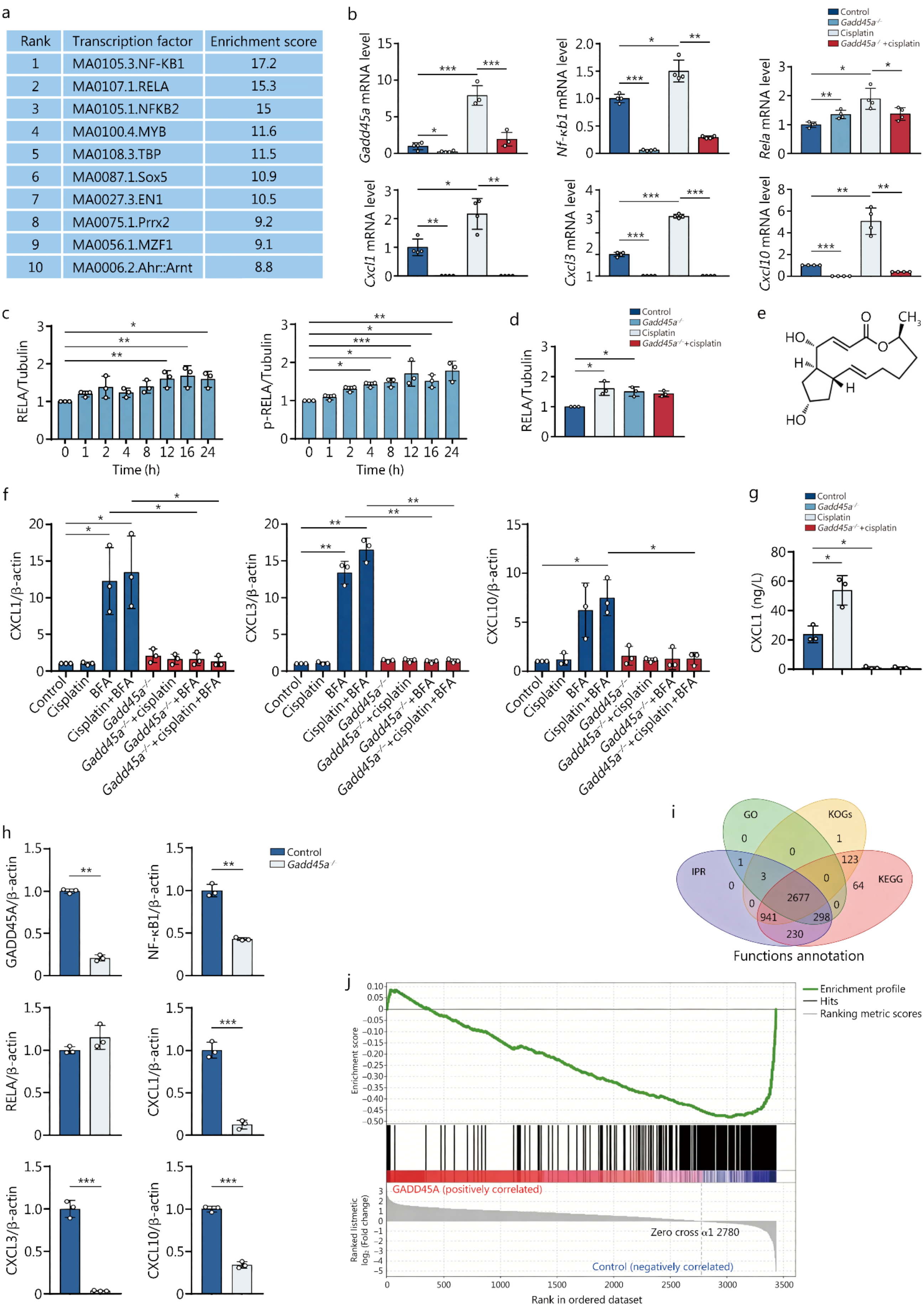

Rank	Transcription factor	Enrichment score
1	MA0105.3.NF-KB1	17.2
2	MA0107.1.RELA	15.3
3	MA0105.1.NFKB2	15
4	MA0100.4.MYB	11.6
5	MA0108.3.TBP	11.5
6	MA0087.1.Sox5	10.9
7	MA0027.3.EN1	10.5
8	MA0075.1.Prrx2	9.2
9	MA0056.1.MZF1	9.1
10	MA0006.2.Ahr::Arnt	8.8

Fig. S3 *Gadd45a* deficiency suppresses NF-κB1-associated CXCL chemokine expression in OC1 cells. **a** Predicted transcription factors shared by CXCL1, CXCL3, and CXCL10, ranked by enrichment score. **b** Relative mRNA expression of *Gadd45a*, *Nf-κb1*, *Rela*, *Cxcl1*, *Cxcl3,* and *Cxcl10* in control and $Gadd45a^{-/-}$ OC1 cells with or without 25 μmol/L cisplatin treatment for 24 h. **c** Quantification of RELA and p-RELA protein levels in OC-1 cells treated with cisplatin for the indicated time points. **d** Quantification of RELA protein expression in control and $Gadd45a^{-/-}$ OC1 cells after cisplatin treatment. **e** Chemical structure of brefeldin A (BFA). **f** Quantification of intracellular CXCL1, CXCL3, and CXCL10 protein levels in control and $Gadd45a^{-/-}$ OC1 cells after treatment with cisplatin and/or BFA. **g** ELISA analysis of secreted CXCL1 levels in the culture supernatants of control and $Gadd45a^{-/-}$ cells with or without cisplatin treatment. **h** Quantification of GADD45A, NF-κB1, RELA, CXCL1, CXCL3, and CXCL10 protein expression in control and $Gadd45a^{-/-}$ OC1 cells. **i** Venn diagram showing the overlap of functional annotation results across different databases. **j** GSEA plot showing reduced enrichment of extracellular protein-associated signatures after *Gadd45a* knockout. $^{*}P<0.05$, $^{**}P<0.01$, $^{***}P<0.001$; horizontal bars indicate the groups compared. NF-κB1. Nuclear factor kappa B subunit 1; RELA. RELA proto-oncogene, NF-κB subunit; NFKB2. nuclear factor κB subunit 2; MYB. MYB proto-oncogene; TBP. TATA-box binding protein; SOX5. SRY-box transcription factor 5; EN1. Engrailed homeobox 1; PRRX2. paired related homeobox 2; MZF1. Myeloid zinc finger 1; AHR. Aryl hydrocarbon receptor; ARNT. Aryl hydrocarbon receptor nuclear translocator; CXCL. C-X-C motif chemokine ligand; BFA. Brefeldin A; GO. Gene Ontology; KOG. EuKaryotic Orthologous Groups; IPR. InterPro; KEGG. Kyoto Encyclopedia of Genes and Genomes; GSEA. Gene set enrichment analysis

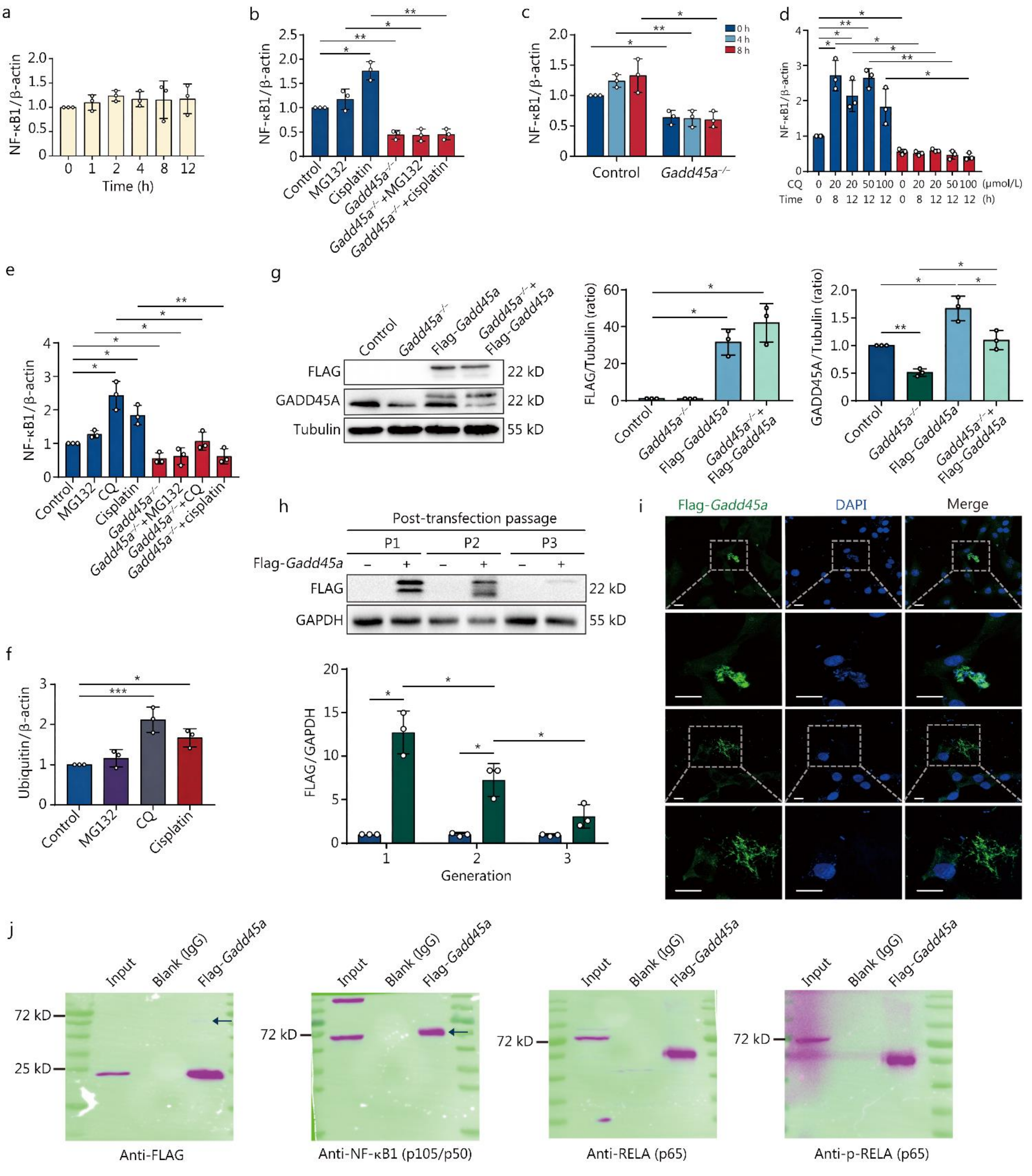

Fig. S4 NF-κB1 stability is mainly regulated through the lysosomal pathway rather than proteasomal degradation. **a** Quantification of NF-κB1 protein expression in OC1 cells treated with 10 μmol/L MG132 for the indicated time points. **b** Quantification of NF-κB1 protein levels in control and *Gadd45a*$^{-/-}$ OC1 cells treated with MG132 and/or cisplatin. **c** Quantification of NF-κB1 expression in control and *Gadd45a*$^{-/-}$ OC1 cells after MG132 treatment for 4 or 8 h. **d** Quantification of NF-κB1 protein expression in control and *Gadd45a*$^{-/-}$ cells treated with different concentrations of chloroquine (CQ) for the indicated time points. **e** Quantification of NF-κB1 protein levels in control and

Gadd45a$^{-/-}$ OC1 cells treated with MG132, CQ, and/or cisplatin. **f** Quantification of ubiquitin protein levels in cells treated with MG132, CQ, and/or cisplatin. **g** Western blotting analysis and quantification of FLAG and GADD45A expression in control and *Gadd45a*$^{-/-}$ OC1 cells after Flag-*Gadd45a* overexpression. **h** Western blotting analysis and quantification of FLAG expression during serial passages of Flag-*Gadd45a*-overexpressing cells. P1-P3 indicate passages 1, 2, and 3. **i** Representative immunofluorescence images showing Flag-*Gadd45a* expression in OC1 cells. Nuclei were counterstained with DAPI. Scale bar=20 μm. **j** Co-immunoprecipitation analysis of Flag-*Gadd45a* with NF-κB1, RELA, and p-RELA. $^{*}P<0.05$, $^{**}P<0.01$, $^{***}P<0.001$; horizontal bars indicate the groups compared. NF-κB1. Nuclear factor κB subunit 1; GADD45A. Growth arrest and DNA damage-inducible alpha; FLAG. FLAG tag; DAPI. 4’6-diamidino-2-phenylindole; GAPDH. Glyceraldehyde-3-phosphate dehydrogenase; CQ. Chloroquine; MG132. Carbobenzoxy-Leu-Leu-leucinal; BFA. Brefeldin A; RELA. RELA proto-oncogene, NF-κB subunit

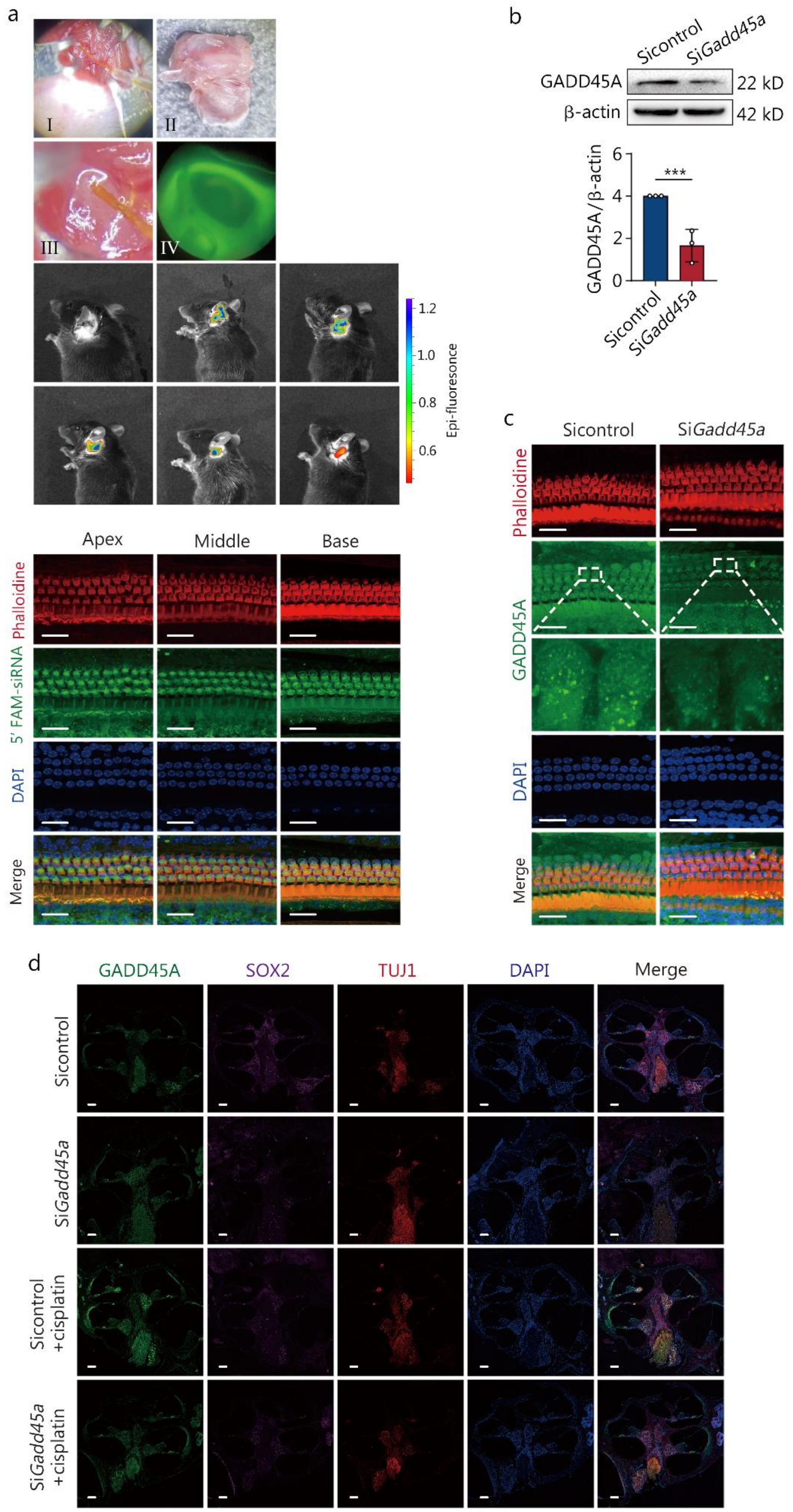

Fig. S5 si*Gadd45a* significantly attenuated the increase of the expression of GADD45A-NF-κB1-CXCL3 axis induced by cisplatin. **a** Schematic illustration of 5'-FAM-siRNA delivery through the

posterior semicircular canal, representative IVIS images showing cochlear distribution at different time points, and representative immunofluorescence images of cochlear hair cells following 5′-FAM-siRNA administration. I. Surgical field of semicircular canal injection. II. Position of the injection hole. III. Connection site of the injection. IV. Fluorescent dye distribution following semicircular canal injection. **b** Representative Western blotting images and quantification of GADD45A expression in cochlear tissues 48 h after siRNA administration. **c** Immunofluorescence staining of GADD45A in the cochlea. **d** Representative immunofluorescence images of cochlear cryosections from mice treated with sicontrol or si*Gadd45a*, with or without cisplatin administration. Scale bar=100 μm. $^{***}P<0.001$; horizontal bars indicate the groups compared. GADD45A. Growth arrest and DNA damage-inducible alpha; DAPI. 4',6-diamidino-2-phenylindole; SOX2. SRY-box transcription factor 2; TUJ1. Class III beta-tubulin; FAM. Fluorescein amidite

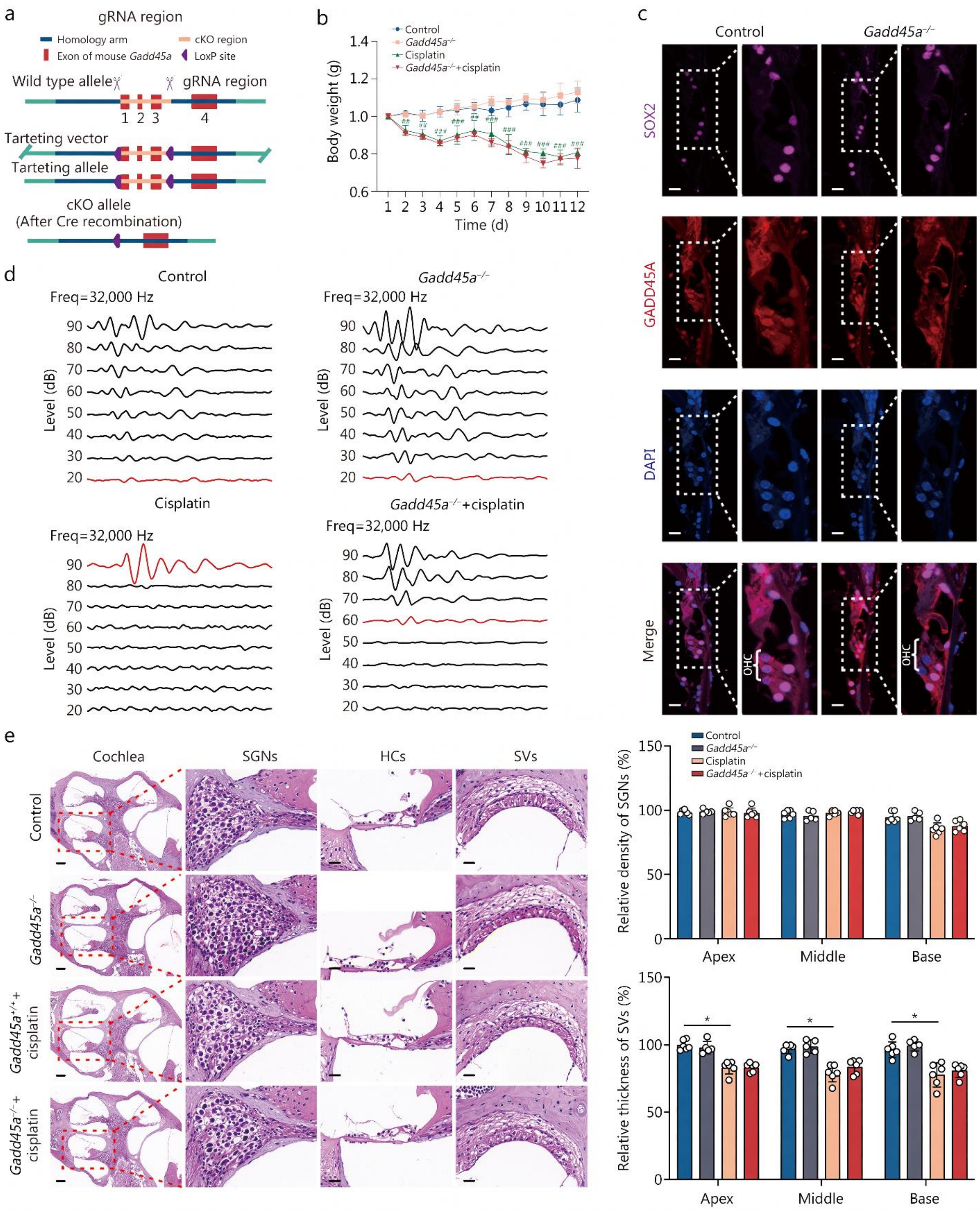

Fig. S6 *Gadd45a* knockout strategy and H&E staining after cisplatin injection in each group. **a** Overview of the targeting strategy for *Gadd45a* knockout. **b** Schematic diagram of body weight changes in mice after saline and cisplatin injection. $^{\#}P<0.01$, $^{\#\#\#}P<0.001$ vs. control group. **c** Immunofluorescence staining showed the conditional *Gadd45a* knockout in hair cells. Scale bar=20 µm. **d** Representative ABR waveform images at 32 kHz for each group. **e** Representative images of H&E staining and quantification of spiral ganglion neurons and stria vascularis thickness in the cochlea.

$^{*}P<0.05$, horizontal bars indicate the groups compared. GADD45A. Growth arrest and DNA damage-inducible alpha; DAPI. 4',6-diamidino-2-phenylindole; SOX2. SRY-box transcription factor 2; SGN. Spiral ganglion neuron; SV. Strial Vascularis; SPL. Sound pressure level
